# Comparative genomics of the extremophile *Cryomyces antarcticus* and other psychrophilic Dothideomycetes

**DOI:** 10.3389/ffunb.2024.1418145

**Published:** 2024-09-06

**Authors:** Sandra V. Gomez-Gutierrrez, Wily R. Sic-Hernandez, Sajeet Haridas, Kurt LaButti, Joanne Eichenberger, Navneet Kaur, Anna Lipzen, Kerrie Barry, Stephen B. Goodwin, Michael Gribskov, Igor V. Grigoriev

**Affiliations:** ^1^ Department of Botany and Plant Pathology, Purdue University, West Lafayette, IN, United States; ^2^ U.S. Department of Energy Joint Genome Institute (JGI), Lawrence Berkeley National Laboratory, Berkeley, CA, United States; ^3^ Crop Production and Pest Control Research Unit, U.S. Department of Agriculture (USDA) - Agricultural Research Service, West Lafayette, IN, United States; ^4^ Department of Biological Sciences, Purdue University, West Lafayette, IN, United States; ^5^ Department of Plant and Microbial Biology, University of California, Berkeley, Berkeley, CA, United States

**Keywords:** extremophile, psychrophile, comparative genomics, *Cryomyces antarcticus*, orthogroups

## Abstract

Over a billion years of fungal evolution has enabled representatives of this kingdom to populate almost all parts of planet Earth and to adapt to some of its most uninhabitable environments including extremes of temperature, salinity, pH, water, light, or other sources of radiation. *Cryomyces antarcticus* is an endolithic fungus that inhabits rock outcrops in Antarctica. It survives extremes of cold, humidity and solar radiation in one of the least habitable environments on Earth. This fungus is unusual because it produces heavily melanized, meristematic growth and is thought to be haploid and asexual. Due to its growth in the most extreme environment, it has been suggested as an organism that could survive on Mars. However, the mechanisms it uses to achieve its extremophilic nature are not known. Comparative genomics can provide clues to the processes underlying biological diversity, evolution, and adaptation. This effort has been greatly facilitated by the 1000 Fungal Genomes project and the JGI MycoCosm portal where sequenced genomes have been assembled into phylogenetic and ecological groups representing different projects, lifestyles, ecologies, and evolutionary histories. Comparative genomics within and between these groups provides insights into fungal adaptations, for example to extreme environmental conditions. Here, we analyze two *Cryomyces* genomes in the context of additional psychrophilic fungi, as well as non-psychrophilic fungi with diverse lifestyles selected from the MycoCosm database. This analysis identifies families of genes that are expanded and contracted in *Cryomyces* and other psychrophiles and may explain their extremophilic lifestyle. Higher GC contents of genes and of bases in the third positions of codons may help to stabilize DNA under extreme conditions. Numerous smaller contigs in *C. antarcticus* suggest the presence of an alternative haplotype that could indicate the sequenced isolate is diploid or dikaryotic. These analyses provide a first step to unraveling the secrets of the extreme lifestyle of *C. antarcticus*.

## 1 Introduction

Life in extreme cold and high-altitude environments like Antarctica and the Alps may be severely restricted, but it is not entirely absent. Under these conditions, rocks are the principal habitat for microorganisms, which are known as epilithic when growing on rock surfaces or endolithic when growing inside the substrate. Endolithic organisms inhabiting rock are categorized into chasmoendoliths, which inhabit fissures and cracks, and cryptoendoliths, which inhabit porous rock substrates (Friedmann, 1982). Cryptoendoliths comprise filamentous fungi, unicellular green algae, and bacteria. Fungi growing under these conditions are known as microcolonial fungi (MCF) or rock-inhabiting fungi (RIF). Most of these organisms belong to the order Dothideomycetes but some are placed in the Eurotiomycetes or remain taxonomically undetermined (Ruibal et al., 2009; Selbmann et al., 2015).

Cryptoendolithic species in genera such as *Cryomyces* and *Friedmanniomyces* have thick, melanized cell walls, produce exopolysaccharides (extracellular polymeric substances, or EPS) outside the hyphae, and have other adaptations to harsh environments (Selbmann et al., 2005). Physiological adaptations such as the production of trehalose and cryoprotectant sugars, polyols, lipids/fatty acids, antifreeze proteins (Robinson, 2001), and hydrolases and oxidoreductases (Duarte et al., 2018; Coleine et al., 2020) play an important role in the adaptation of these organisms to cold. In general, psychrophilic black fungi show increased protein expression when grown at 1 °C; *Friedmanniomyces endolithicus*, one of the species included in this work, showed increased numbers of high-molecular-weight proteins in comparison to a mesophilic reference species (Tesei et al., 2012), suggesting substantial reprogramming of protein expression at low versus high temperatures. These and other physiological adaptations to extreme environments should be reflected in genome modifications.

The production of proteins and enzymes underlies the physiological mechanism of cold tolerance in fungi. Observed changes occur in both gene regulation and as mutational changes to individual proteins. One of the simplest adaptations is the expansion or contraction of paralogous groups. Changes in gene number provide a rapid means to modify proteome composition, enzymatic activity, and the constituents of the membrane, all of which are important in cold/stress adaptation. However, these mechanisms are complex and not fully understood (Maggi et al., 2013).

Adaptation to cold environments may require the alteration of proteins such as those related to membrane transport and lipid biosynthesis to maintain membrane flexibility at low temperatures. A proteome comparison can identify proteins that are significantly altered in cold-adapted fungi. However, studies of proteome composition mostly have been limited to the comparison of only a few species and conditions at a time (Tesei et al., 2012), limiting our ability to see general proteomic patterns related to adaptation and survival in extreme conditions. Identification of such protein and enzyme patterns in the proteomes of cold-adapted fungi can identify proteins that are responsible for physiological mechanisms of cold tolerance.

Expansion or contraction of orthologous protein families is one of the simplest ways for organisms to adapt to an extreme environment. Expansion of specific groups has been identified in many species (Su et al., 2016; Stajich, 2017; Paiva et al., 2024). Previous analysis of a draft genome of *C. antarcticus*, sequenced with Ion PI, identified little that stood out, causing the authors to conclude there was “nothing special in the specialist” (Sterflinger et al., 2014). However, that analysis included comparisons with only five other species, only three of which were in the Dothideomycetes, and the assembly was very fragmented. In this work, we compare the proteome composition of 52 species in the class Dothideomycetes, 19 of which are extremophiles (cold, acidophilic, ethanol vapor, xerophilic, or salt tolerant). The selected species include 11 psychrophiles which are the primary targets of our analysis. The non-psychrophilic species include lichens, yeasts, parasitic species, opportunistic animal pathogens, and plant pathogens from selected Dothideomycetes groups. We examine and compare the expansion and contraction of orthologous groups to test the hypothesis that families of genes associated with adaptation may be altered in these stress-tolerant Dothideomycetes.

Based on previously published work (reviewed by Gostinčar et al., 2023), we expect to see changes in genes related to pigment production, cell wall biosynthesis, membrane transporters, production of compatible solutes (such as glycerol), fatty acid synthesis, and response to reactive oxygen species (ROS). In addition, specific groups necessary for survival in other environments, for instance genes involved in lignin degradation and carbohydrate‐active enzymes (CAZymes) required for pathogenicity to plants, may be dispensable in environments where the corresponding substrates are unavailable. By synthesizing results across a broad range of species, we hope to identify general principles of adaptation to extremely cold environments and determine whether these mechanisms are also found in fungal species adapted to other types of stresses.

## 2 Materials and methods

### 2.1 Genome sequencing, assembly, and annotation

*Cryomyces antarcticus* isolate 116301 was obtained from the CBS culture collection (Utrecht, the Netherlands). The fungus was cultured on 2% potato dextrose agar plates at 4 C for more than 6 months. Mycelia were scraped off the surface of the plates and lyophilized. DNA was extracted using Qiagen’s DNeasy kit and RNA was extracted using the RNeasy kit. Its genome was sequenced using the PacBio platform. For the PacBio library, 5 μg of genomic DNA was sheared to >10 kb using Covaris g-Tubes. The sheared DNA was treated with exonuclease to remove single-stranded ends followed by end repair and ligation of blunt adapters using the SMRTbell Template Prep Kit 1.0 (Pacific Biosciences). The library was purified with AMPure PB beads. PacBio Sequencing primer was then annealed to the SMRTbell template library and sequencing polymerase was bound to them using the Sequel II Binding kit 1.0. The prepared SMRTbell template libraries were then sequenced on a Pacific Biosystems Sequel II sequencer using 8M v1 SMRT cells and Version 1.0 sequencing chemistry with 1×900 min sequencing movie run times. Filtered subread data were processed with the JGI QC pipeline to remove artifacts and organellar reads. Filtered Circular Consensus Sequence (CCS) reads were assembled with Flye version 2.7.1-b1590 (Kolmogorov et al., 2019) to generate an assembly and polished with the PacBio SMRTLINK (v8.0.0.80529) using the gcpp –algorithm arrow command.

For the *Cryomyces antarcticus* transcriptome, plate-based RNA sample prep was performed using the PerkinElmer Sciclone NGS robotic liquid handling system and Illumina’s TruSeq Stranded mRNA HT sample prep kit, using poly-A selection of mRNA following the protocol outlined by Illumina in their user guide (https://support.illumina.com/sequencing/sequencing_kits/truseq-stranded-mrna.html) and with the following conditions: total RNA starting material was 1 μg per sample and 8 cycles of PCR were used for library amplification. The prepared library was then quantified using KAPA Biosystem’s next-generation sequencing library qPCR kit (Roche) and run on a Roche LightCycler 480 real-time PCR instrument. The quantified library was then multiplexed with other libraries, and the pool of libraries was prepared for sequencing on the Illumina NovaSeq 6000 sequencing platform using NovaSeq XP v1 reagent kits, S4 flow cell, following a 2×150 indexed run recipe. Raw RNAseq reads were filtered and trimmed using the BBDuk kmer matching (kmer=25) and phred trimming method set at Q6. Following trimming, reads under the length threshold were removed (minimum length of 25 bases or 1/3 of the original read length - whichever is longer). Filtered reads were used as input for *de novo* assembly of RNA contigs using Trinity version 2.8.5 with parameters –normalize_reads and –jaccard_clip.

The genome was annotated using the JGI Annotation pipeline and made available at the JGI MycoCosm genome portal (http://mycocosm.jgi.doe.gov/Cryan3) along with tools for interactive comparative analysis (Grigoriev et al., 2014). Both the assembly and annotation have been deposited to GenBank under accession JBDRTV000000000.

### 2.2 Acquisition of additional genome data

The proteomes of 52 fungal species were obtained from the JGI MycoCosm portal (https://mycocosm.jgi.doe.gov/mycocosm/; Grigoriev et al., 2014) (**Table 1**). The majority of the species belong to the subclass Dothideomycetidae within the Dothideomycetes. Members of this subclass were chosen for their close phylogenetic relationship to *Cryomyces antarcticus* and *C. minteri*, as indicated by the phylogenetic tree available in MycoCosm (https://mycocosm.jgi.doe.gov/mycocosm/species-tree/tree;j7jS2o?organism=dothideomycetes). The group of psychrophilic fungi (https://mycocosm.jgi.doe.gov/Psychrophilic_fungi) consists of 11 species, including our target species *C*. *antarcticus* and *C. minteri*, as well as the species *Aureobasidium subglaciare, Extremus antarcticus, Friedmanniomyces simplex, F. endolithicus, Hortaea thailandica, Rachicladosporium* sp., *Rachicladosporium antarcticum*, and two isolates of the genus *Alternaria* from the order Pleosporales, *Alternaria* sp. UNIPAMPA012 and *Alternaria* sp. UNIPAMPA017. Both these species were isolated from healthy leaves of Antarctic hair grass (https://mycocosm.jgi.doe.gov/Altsp012_1/Altsp012_1.home.html).

**Table 1:** Summary information about the Dothideomycetes fungal species included in the comparative genomic analyses.

Species	JGI Portal Id	Lifestyle traits	Project citation
*Acarospora strigata*	Acastr1	Lichen	McDonald et al., 2013
*Acidomyces richmondensis* BFW	Aciri1	Acidophilic	Mosier et al., 2016
*Alternaria* sp. UNIPAMPA012	Altsp012_1	Psychrophilic; endophytic	With permission from Dr. Francis Martin
*Alternaria* sp. UNIPAMPA017	Altsp017_1	Psychrophilic; endophytic	
*Aureobasidium namibiae*	Aurpu_var_nam1	Heat tolerant	Gostinčar et al., 2014
*Aureobasidium subglaciare*	Aurpu_var_sub1	Psychrophilic	
*Baudoinia compniacensis*	Bauco1	Ethanol vapor tolerant	Ohm et al., 2012
*Capnodiales* sp. MNA-CCFEE 6580	Cap6580	Endolithic	With permission from Dr. Laura Selbmann
*Cercospora berteroae*	Cerbe1	Plant pathogen	de Jonge et al., 2018
*Cercospora zeae-maydis*	Cerzm1	Plant pathogen	Haridas et al., 2020
*Cladonia grayi*	Clagr3	Lichen	Armaleo et al., 2019
*Cladosporium sphaerospermum* UM 843	Clasph1	Halotolerant	Ng et al., 2012
*Cladosporium fulvum*	Clafu1	Plant pathogen	de Wit et al., 2012
*Coniosporium apollinis*	Conap1	Epilithic black yeast	Teixeira et al., 2017
*Cryomyces antarcticus*	Cryan3	Psychrophilic; endolithic	This study
*Cryomyces minteri* CCFEE 5187	Crymi1	Psychrophilic; endolithic	Coleine et al., 2020
*Delphinella strobiligena*	Delst1	Yeast-like fungus	Haridas et al., 2020
*Dibaeis baeomyces*	Dibbae1	Lichen	McDonald et al., 2013
*Dissoconium aciculare*	Disac1	Mycoparasitic	Haridas et al., 2020
*Dothistroma septosporum*	Dotse1	Plant pathogen	de Wit et al., 2012
*Elsinoë ampelina*	Elsamp1	Plant pathogen	Haridas et al., 2020
*Extremus antarcticus* MNA-CCFEE 451	Extant1	Psychrophilic; endolithic	With permission from Dr. Laura Selbmann
*Friedmanniomyces endolithicus* CCFEE 5311	Frien1	Psychrophilic; endolithic	Coleine et al., 2020
*Friedmanniomyces simplex* CCFEE 5184	Frisi1	Psychrophilic; endolithic	Coleine et al., 2020
*Glonium stellatum*	Glost2	Opportunistic	Peter et al., 2016
*Graphis scripta*	Grascr1	Lichen	McDonald et al., 2013
*Hortaea acidophila*	Horac1	Acidophilic	Haridas et al., 2020
*Hortaea werneckii*	Horwer1	Halotolerant	Lenassi et al., 2013
*Hortaea thailandica*	Horth1	Psychrophilic	Coleine et al., 2020
*Lasallia pustulata* (*Umnilicaria pustulata*)	Umbpus1	Lichen	Dal Grande et al., 2017
*Lobaria pulmonaria*	Lobpul1	Lichen	With permission from Dr. Ólafur Andrésson
*Myriangiaceae* sp. NC1570	Myrian1	Endophytic	With permission from Dr. Elizabeth Arnold
*Myriangium duriaei*	Myrdu1	Parasitic	Haridas et al., 2020
*Piedraia hortae* CBS 480.64 v1.1	Pieho1_1	Opportunistic	Haridas et al., 2020
*Polychaeton citri*	Polci1	Opportunistic	Haridas et al., 2020
*Pseudocercospora eumusae* ^1^	Myceu1	Plant pathogen	Chang et al., 2016
*Pseudocercospora fijiensis* ^2^	Mfijiensis_v2	Plant pathogen	Arango Isaza et al., 2016
*Pseudocercospora musae*	Psemus1	Plant pathogen	Chang et al., 2016
*Pseudocercospora ulei*	Pseule1	Plant pathogen	With permission from Dr. Eduardo Mizubuti
*Rachicladosporium* sp. CCFEE 5018	Rac5018_1	Psychrophilic, endolithic	Coleine et al., 2017
*Rachicladosporium antarcticum* CCFEE 5527	Racan1	Psychrophilic, endolithic	Coleine et al., 2017
*Sphaerulina musiva* ^4^	Sepmu1	Plant pathogen	Ohm et al., 2012
*Sphaerulina populicola* ^5^	Seppo1	Plant pathogen	Ohm et al., 2012
*Teratosphaeriaceae* sp. NC1134	TerNC1134_1	Endophytic	With permission from Dr. Elizabeth Arnold
*Teratosphaeria nubilosa*	Ternu1	Plant pathogen	Haridas et al., 2020
*Viridothelium virens*	Tryvi1	Lichen	Haridas et al., 2020
*Xylona heveae*	Xylhe	Endophytic	Gazis et al., 2016
*Zasmidium cellare*	Zasce1	Ethanol vapor tolerant	Haridas et al., 2020
*Zasmidium cellare*	Zymar1	Plant pathogen	Stukenbrock et al., 2012
*Zymoseptoria brevis*	Zymbr1	Plant pathogen	Grandaubert et al., 2015
*Zymoseptoria pseudotritici*	Zymps1	Plant pathogen	Stukenbrock et al., 2012
*Zymoseptoria tritici* ^3^	Zymtr1	Plant pathogen	Goodwin et al., 2011

^1^Former name: *Mycosphaerella eumusae*.

^2^Former name: *Mycosphaerella fijiensis*.

^3^Former name: Mycosphaerella graminicola.

^4^Former name: *Septoria musiva*.

^5^Former name: *Septoria populicola*.

Among the selected species, we include representatives of different lifestyles: endophytic, endolithic, molds, yeasts, opportunistic human pathogens, parasites, and plant pathogens (**Table 1**). The non-psychrophilic group contains fungal pathogens of a diverse range of hosts including the sugar beet pathogen *Cercospora berteroae*, the corn pathogen *Cercospora zeae-maydis*, the tomato and grape pathogens *Cladosporium fulvum* and *Elsinoë ampelina*, the banana pathogens *Pseudocercospora* (formerly *Mycosphaerella*) *eumusae*, *P*. (*Mycosphaerella*) *fijiensis*, and *P. musae*, the wheat pathogen *Zymoseptoria tritici* (formerly *Mycosphaerella graminicola*) and its close relatives *Z. ardabiliae*, *Z. brevis*, and *Z. pseudotritici* (isolated from wild grass species). We also included the forest pathogens *Dothistroma septosporum, Pseudocercospora ulei, Sphaerulina musiva* (formerly *Septoria musiva*; teleomorph: *Mycosphaerella populorum*)*, Sphaerulina populicola* (formerly *Septoria populicola*), and *Teratosphaeria nubilosa*. In addition, other extremophiles are included in the analysis to compare the survival strategies under different extreme environmental conditions: the acidophilic fungi *Acidomyces richmondensis* and *Hortaea acidophila;* the heat-tolerant *Aureobasidium namibiae;* the ethanol vapor and heat-tolerant species *Baudoinia compniacensis* and *Zasmidium cellare*; and the salt-tolerant species *Hortaea werneckii* and *Cladosporium sphaerospermum*. Furthermore, six species of lichens (*Acarospora strigata*, *Cladonia grayi*, *Dibaeis baeomyces, Graphis scripta, Umbilicaria pustulata* and *Lobaria pulmonaria*) from the class Lecanoromycetes, and one endophytic species (*Xylona heveae*) from the Xylonomycetes, were included as outgroups.

The genome sizes of fungal species in the study were calculated, and an ANOVA test was conducted using the “car” package in R (Fox and Weisberg, 2019). To determine statistically significant differences between the genome sizes of fungal groups, Tukey’s test was subsequently performed using the “stats” base package in R (R Core Team, 2024).

### 2.3 GC contents of the genomes, coding sequences and third-codon position

GC content was analyzed using the stats.sh function of bbtools (Bushnell, 2014) for determining the overall GC contents of the whole-genome sequences, and the EMBOSS application “cusp” (Rice et al., 2000) for calculating the GC content of protein-coding genes and the third-codon position (GC3). GC contents of genomic contigs and scaffolds of *C. antarcticus* and *C. minteri* were calculated using countgc.sh of the bbtools package (Bushnell, 2014). Plots representing the GC distribution were created using ggplot2 (Wickham, 2016) in R. Statistically significant differences in genome-wide GC content, GC3 and GC content of protein-coding genes between the groups of fungi were studied with a MANOVA test using the “car” (Fox and Weisberg, 2019) package in R. Tukey’s test was conducted for multiple group comparison using the base R “stats” package.

### 2.4 Genome-wide analysis of repeat-induced point mutation

Genome assemblies of species included in this study were used to conduct RIP analyses using the RIPper software (https://github.com/TheRIPper-Fungi/TheRIPper/) (van Wyk et al., 2019). This program calculates the RIP indices and the average GC contents of whole-genome sequence information using a sliding-window approach. Large RIP-affected genomic regions (LRARs) and the index values associated with them are also determined. We used the default parameters for genome-wide RIP index calculations consisting of 1000-bp windows with a 500-bp step size. Stringent parameters were used (product index value > 1.15; substrate index value < 0.75; RIP composite index > 0) to identify genomic regions affected by RIP. RIPper considers a window as RIP positive if all three indices indicate RIP activity. Only regions of more than 4000 consecutive bp that are affected by RIP are considered as LRARs (van Wyk et al., 2019). The total estimated genome-wide RIP percentage and the composite index for LRARs are shown along with a rooted species tree that was constructed using OrthoFinder v. 2.5.5 (Emms and Kelly, 2019) and the proteome files of each species. The species phylogenetic tree along with the RIP analysis results were visualized using the Interactive Tree of Life (iTOL v. 5.0; Letunic and Bork, 2021).

### 2.5 Identification of orthogroups

OrthoFinder v. 2.5.5 (Emms and Kelly, 2019) was used to identify orthologous gene families using DIAMOND (Buchfink et al., 2021) and MCL graph clustering (Enright et al., 2002) for sequence comparison and STAG (Emms and Kelly, 2018) as the default species tree method. For each of the 25,761 orthogroups, gene counts per species were normalized by converting to standard normal deviates using the 50% trimmed mean and standard deviation of each orthogroup. Expanded and contracted orthogroups were identified by comparing the normalized scores for the 11 psychrophiles and the 41 non-psychrophiles using Welch’s t-test statistic,


$$\text{t=}\frac{c_{p}-{\text{c}}_{\text{n}}}{\sqrt{{\text{s}}_{\text{p}}^{2}+{\text{s}}_{\text{n}}^{2}}}$$

where c is the mean normalized count, s is the standard deviation, and n is the number of sequences. Subscripts *p* and *n* indicate psychrophiles and non-psychrophiles, respectively. The 50 most expanded and 50 most contracted groups were selected for further analysis.

### 2.6 Correlational matrix and heatmap

The R built-in stats functions “cor” (R Core Team, 2023) and “reshape2” (Wickham, 2007) packages were used to build a correlation matrix using the normalized counts. We used the package “factoextra” (Kassambara and Mundt, 2020) in R to conduct a principal component analysis (PCA) using the correlational matrix to determine clusters of species explained by the orthogroups. The packages “ggplot2” and “ggcorrplot” (R Core Team, 2023) were used to visualize the associations in the matrix.

The R package *pheatmap* (v1.0.12) (Kolde, 2019) was used to construct a heatmap of the selected expanded and contracted orthogroups. Correlation was used as the distance measure for both orthogroups and species, and groups were clustered using Ward’s method (Ward Jr., 1963).

### 2.7 Functional annotation of orthogroups

Orthogroup annotations were obtained by Blast comparison to the UniRef50 database (The UniProt Consortium, 2023) with a maximum E-value of 10^−10^ and minimum of 40% identity. To identify domain annotations and GO terms associated with orthogroups, we used Interproscan (v 5.66-98.0) and InterPro (v 98.0) (Paysan-Lafosse et al., 2023). CAZymes were annotated using dbCAN2 (Yin et al., 2012).

## 3 Results

### 3.1 Genome features of psychrophilic fungi

**Figure 1:**
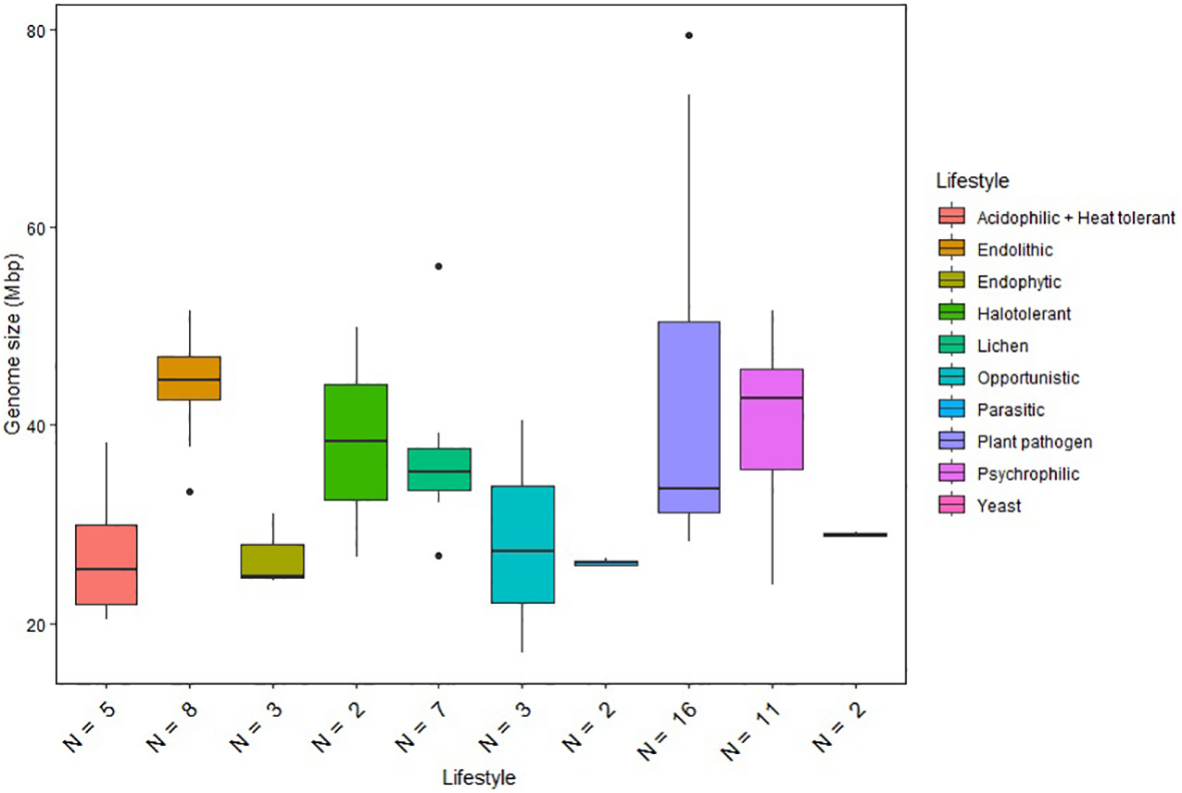
Genome size distribution of fungi with different lifestyles. Horizontal lines indicate the means, boxes show the group sizes, the vertical lines represent the range of the data, and the dots are outliers. Lifestyle groups are color coded according to the legend on the right. Seven fungal species that belong to both endolithic and psychrophilic lifestyles are represented in both groups in the figure. The lifestyle Acidophilic + Heat tolerant encompasses the two ethanol vapor-tolerant fungi included in the study.

**Figure 2:**
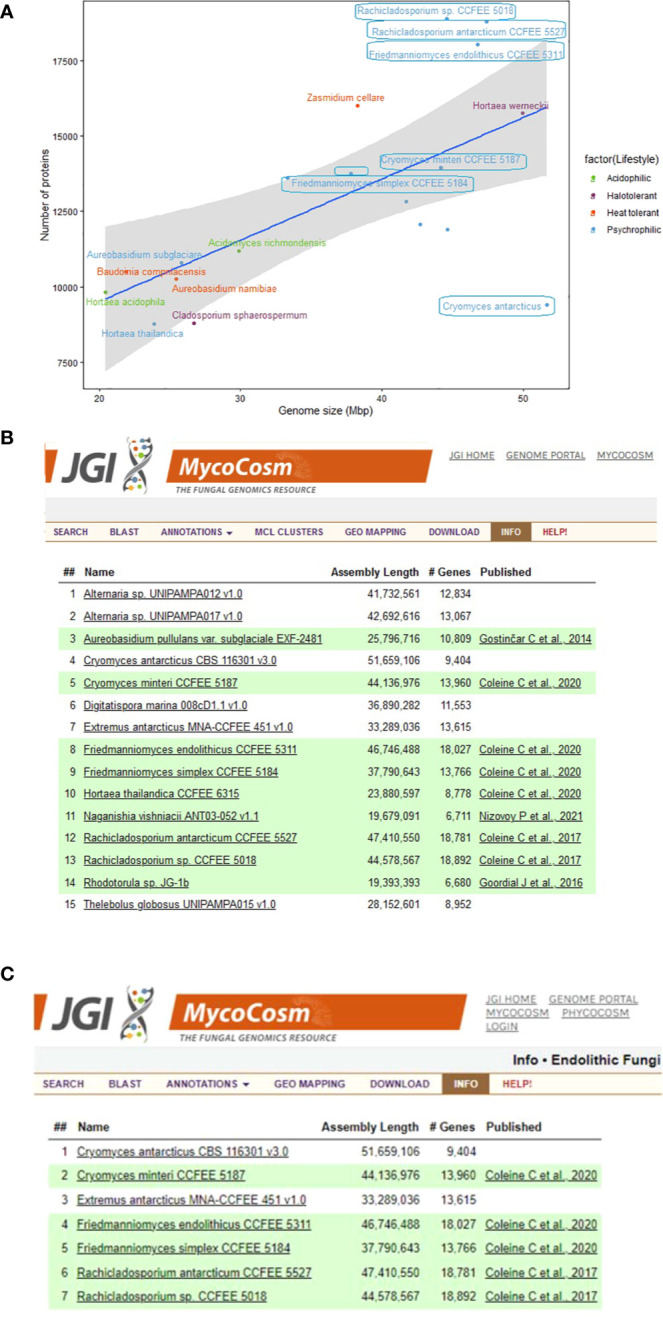
Summary information about the genomes of extremophilic fungi in MycoCosm, **(A)** Genome sizes compared to the numbers of encoded proteins for the extremophilic fungi. Each group is color coded according to the legend on the right. Species framed indicate the psychrophiles that are also endolithic. **(B, C)** MycoCosm group pages for for **(B)** psychrophilic (https://mycocosm.jgi.doe.gov/Psychrophilic_fungi) and **(C)** endolithic (https://mycocosm.jgi.doe.gov/Endolithic_fungi) groups of fungi with sequenced and annotated genomes showing genome assembly sizes, gene counts, and genome publication status.

**Figure 3:**
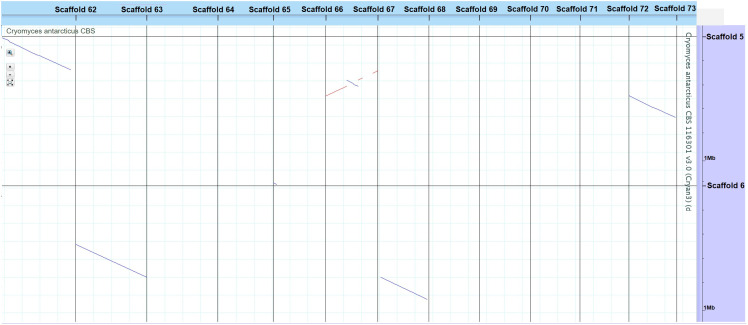
VISTA dot plot of selected *Cryomyces antarcticus* contigs from the whole-genome DNA-based self alignment (Dubchak, 2007) – see **Supplementary Figure 1** for complete picture. The red and blue diagonal lines are nucleotide matches between different contigs, suggesting DNA duplication.

Genome sizes of the psychrophilic fungi range from 23.88 to 51.66 Mbp, with *C. antarcticus* being the largest in the list (**Figure 1**). The number of encoded proteins varies from 8,778 to 18,892, with 9,404 in *C. antarcticus* (**Figure 2**). Repeat content in the two *Cryomyces* species varied from 8.4% in *C. minteri* to 12.5% in C. *antarcticus*, or about 2 Mbp, which also helps to explain the disparity in genome sizes between these species. A VISTA dot plot of the *C. antarcticus* contigs revealed numerous diagonal lines (**Figure 3**; **Supplementary Figure 1**) indicating that the sequenced isolate may be a polymorphic dikaryon or possibly a diploid. The VISTA dot plot displays blue and red lines, which represent off-diagonal matching contigs or their parts, indicating a partially duplicated genome (**Supplementary Figure 1**). Scaffold 6 comprises entire scaffolds 62 and 67, while scaffold 5 is composed of scaffold 61 (label not shown but it precedes 62), inverted scaffold 66, and scaffold 72 (**Figure 3**).

To gain additional insights into the likely diploidy or possible dikaryotic ploidy of *C. antarcticus*, we generated a k-mer plot using a k-mer length of 31 (**Supplementary Figure 2**). The k-mer frequency plot reveals two peaks, providing further evidence of diploidy or dikaryosis, or even a recent whole-genome duplication. The taller peak indicated by a long arrow represents k-mers found at a higher frequency (**Supplementary Figure 2**), common to both chromosome sets. The second, smaller peak indicated by a small arrow contains k-mers that are unique to one of the homologous chromosomes or have a lower frequency because they are not duplicated across the homologous chromosomes (Ranallo-Benavidez et al., 2020). The small peaks observed in the k-mer frequency plot normally indicate repeats in the genome (Lo and Chain, 2014).

We also compared the genome sizes of the psychrophilic species with those of other lifestyle groups included in our analysis (**Figure 1**). The comparison revealed significant variations in genome size within individual lifestyles. Plant pathogens generally exhibit the largest genome sizes among the Dothideomycetes species included in the study. Notably, psychrophilic (including endolithic) and halotolerant fungi exhibit similar ranges of genome sizes, while other extremophiles classified as acidophilic and heat tolerant have reduced genome sizes compared to the other fungi. Although the ANOVA test revealed significant differences between the lifestyle groups, Tukey’s test did not find any statistically significant differences between specific pairs of lifestyle groups. This suggests that, while there is an overall significant difference between groups, the specific pairwise differences are not strong enough to be considered significant after adjusting for multiple comparisons. Therefore, genome size appears not to be a reliable predictor of a given lifestyle and cannot be used to differentiate psychrophilic fungi from other groups.

There is a positive correlation between genome size and the number of encoded proteins among extremophilic fungi (**Figure 2**), where larger genome sizes corresponded to higher protein counts. However, there was a departure from this trend for six psychrophilic fungi, which deviated from the linear regression line. Notably, *F. endolithicus, Rachicladosporium* sp., and *R. antarcticum* showed higher-than-expected protein counts relative to their genome sizes, while *C. antarcticus* showed lower-than-expected protein counts. Despite this difference, we did not observe a consistent pattern for genome/proteome ratio among all psychrophilic or extremophilic fungi when compared to those with other lifestyles. This suggests that the discrepancies in genome sizes compared to proteome size for some psychrophilic fungi may be unique to the individual species rather than indicative of general mechanisms of adaptation to extreme environments.

### 3.2 GC contents in *Cryomyces* genomes

**Figure 4:**
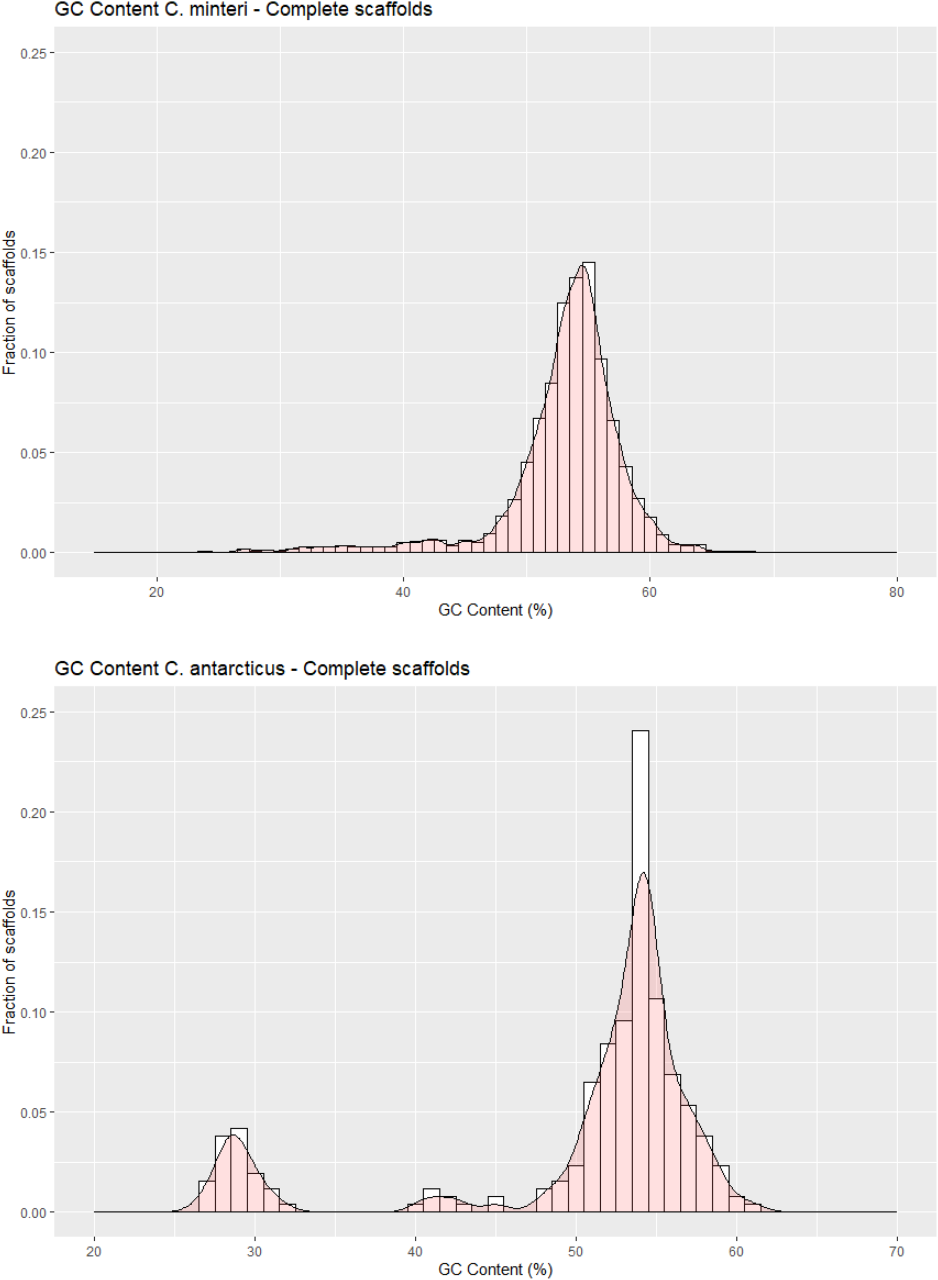
GC contents in scaffolds of **(A)** *Cryomyces minteri* and **(B)** *C. antarcticus.* The y axis “Fraction of scaffolds” represents the fraction of the total complete scaffolds in each species that have a given GC content.

**Figure 5:**
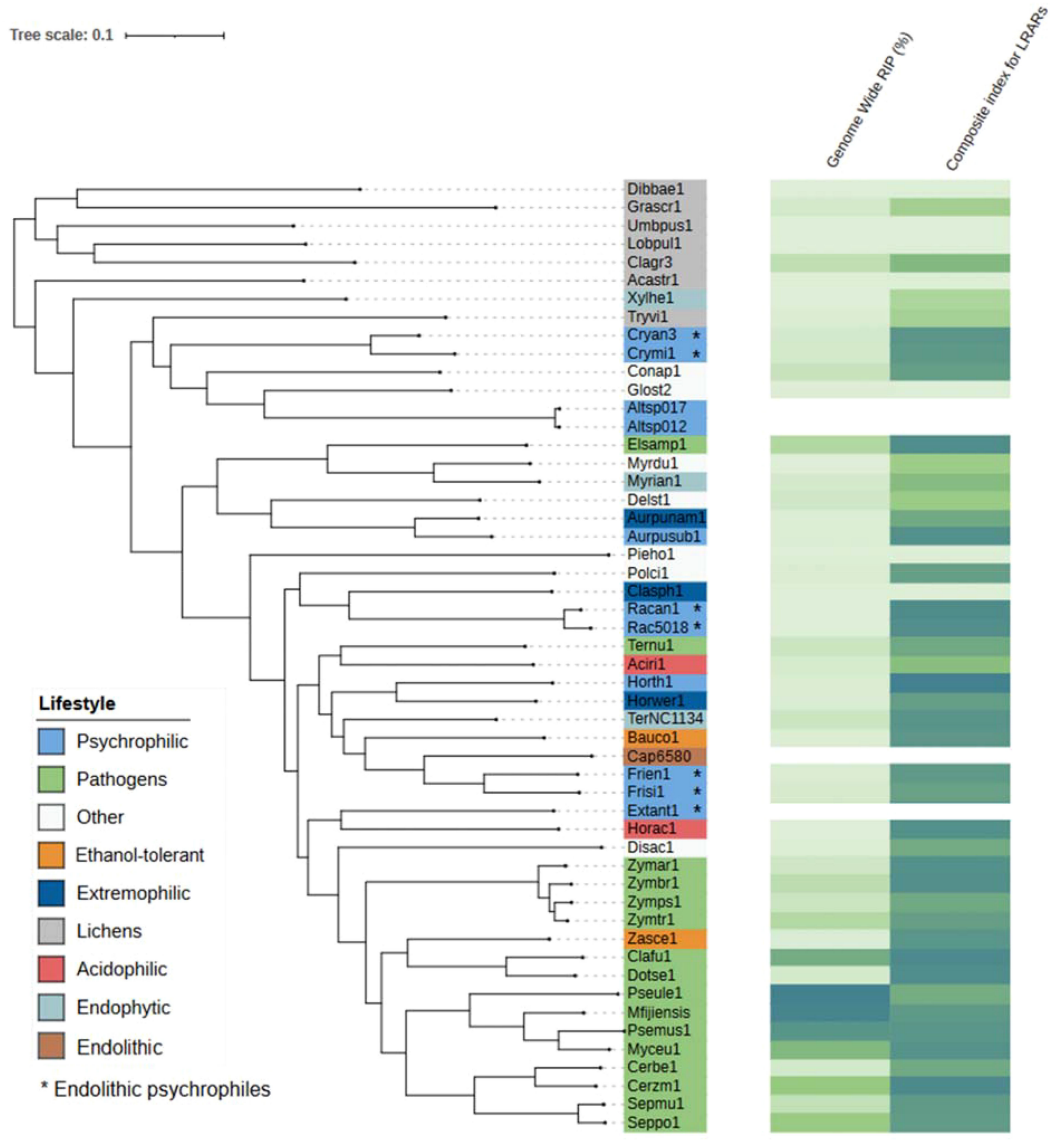
Phylogenetic tree produced by OrthoFinder showing the percentage of each genome affected by RIP and the composite index for large RIP-affected genomic regions (LRARs) in each species. Tree leaves are labeled by JGI portal ids (see **Table 1** for full species names). Extremophilic classification encompasses heat tolerant + halotolerant fungi.

The *C. antarcticus* genome exhibited a bimodal distribution of GC content across contigs (**Figure 4**). The main peak of GC content contains a higher proportion of contigs with GC content values between 42% and 60%, while a small peak of low-GC content between 0% and 32% contains a smaller number of contigs. This may be associated with some residual contamination or the presence of RIP mutations in repetitive regions, giving rise to low GC content. In *C. minteri*, we did not observe a clear bi-modal distribution of GC content as we did for *C. antarcticus*, potentially reflecting its lower proportion of repetitive DNA. We then analyzed the genome-wide RIP mutations in the genomes and found that both *C. minteri* and *C. antarcticus* showed a similar percentage of genome-wide RIP mutations of 4.36% and 4.04%, respectively, as calculated with the RIPper tool (**Figure 5**, **Supplementary Table 1**). These values are the highest for total estimated genome-wide RIP among the psychrophilic species in this study. Taken together, these results suggest that the genomes of these two psychrophilic species might have been affected by RIP to some extent and at a higher level than other psychrophilic fungi. However, once again we did not observe a consistent pattern of genome-wide RIP or associated metrics across all psychrophilic fungal genomes. This suggests that this mechanism may not represent a general strategy for adaptation to cold stress.

### 3.3 GC content in whole-genome, protein-coding regions and GC3 is higher in psychrophilic and extremophilic fungi

**Figure 6:**
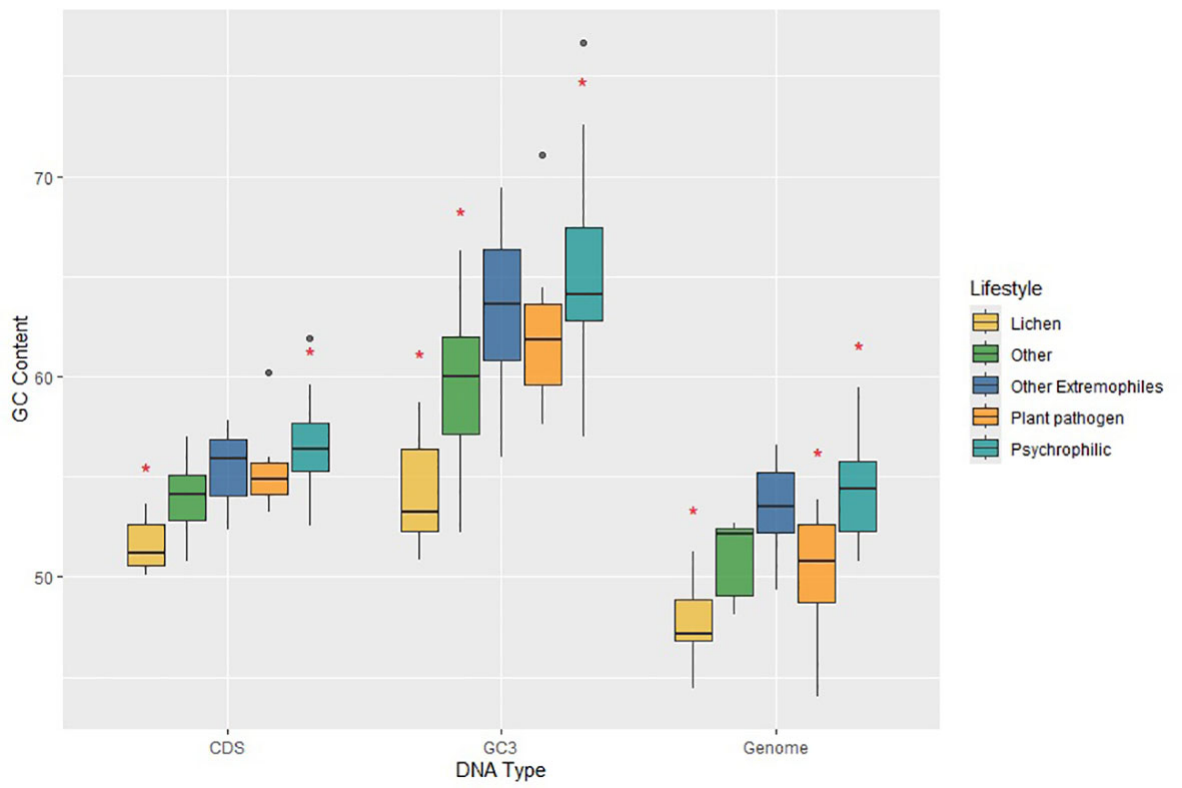
Plot of GC contents for coding sequences (CDS), third-position bases of genes (GC3) and the total genomes (Genome) for fungi in the different lifestyle groups, which are colored according to the legend on the right. Horizontal lines indicate the means, boxes show the group sizes, the vertical lines represent the range of the data, and the dots are outliers. Red star symbols denote groups with statistically significant differences as determined by Tukey’s HSD test. Lifestyle groups are color coded according to the legend on the right. The category ‘Other Extremophiles’ includes acidophilic, heat tolerant and halotolerant fungi.

We grouped extremophilic fungi, excluding psychrophiles, into a single category encompassing acidophilic, heat-tolerant, and halotolerant fungi. Next, we examined whether significant variations exist in GC contents across whole genomes, protein-coding regions, and GC content in the third-codon position (GC3) between psychrophilic fungi and other fungal groups. The overall GC content in *C. antarcticus* and *C. minteri* is 53.31%. and 53.89%, respectively. While these values are consistent with those of fungi with other lifestyles, a broader analysis reveals some trends. Specifically, when considering the entire group of psychrophilic and extremophilic fungi, we observed that GC content in the psychrophiles (average = 54.38%) and extremophiles (average=53.47%) is higher than that in plant pathogens (average=50.38%), lichens (average = 47.74%) and fungi with other lifestyles (average = 51.02%). The MANOVA test found significant differences in genome-wide GC content, GC3 and GC content of coding sequences. Tukey’s test revealed significant differences in the GC content of the whole genome, with psychrophiles exhibiting higher GC content compared to plant pathogens and lichens, and the group of other extremophiles also showing significant differences compared to lichens (**Figure 6**). Further examination using Tukey’s test revealed that the GC contents of coding sequences and the GC3 in psychrophilic fungi are significantly higher than those in lichens, opportunistic fungi, and endophytic fungi, with the latter two categorized as “other” (**Figure 6**). For extremophiles, significant differences were found in GC contents of coding sequences and GC3 only compared to lichens (**Figure 6**). Overall, these results suggest that the genome-wide GC content may be a distinctive feature in psychrophilic fungi, potentially contributing to their mechanisms for tolerance to cold environments, as indicated by significant differences observed compared to those for plant pathogens and lichens.

### 3.4 OrthoFinder results

OrthoFinder assigned 616,448 genes from 52 species to 25,761 orthogroups. The number of orthogroups with all species present was 1,968, revealing the core genome among the tested fungal species. We identified 40 species-specific orthogroups for *C. antarcticus*, all of which have at least 2 paralogs in the species, and one orthogroup that has 4 members in *C. antarcticus*. In the case of *C. minteri*, we identified 148 species-specific genes, all of which are present in at least 2 copies, and in some cases even 4 to 8 copies. Overall, we found 233 genus-specific orthogroups in *Cryomyces*.

### 3.5 Expansion and contraction events in gene families distinguish psychrophilic and extremophilic fungi from those with other lifestyles

**Figure 7:**
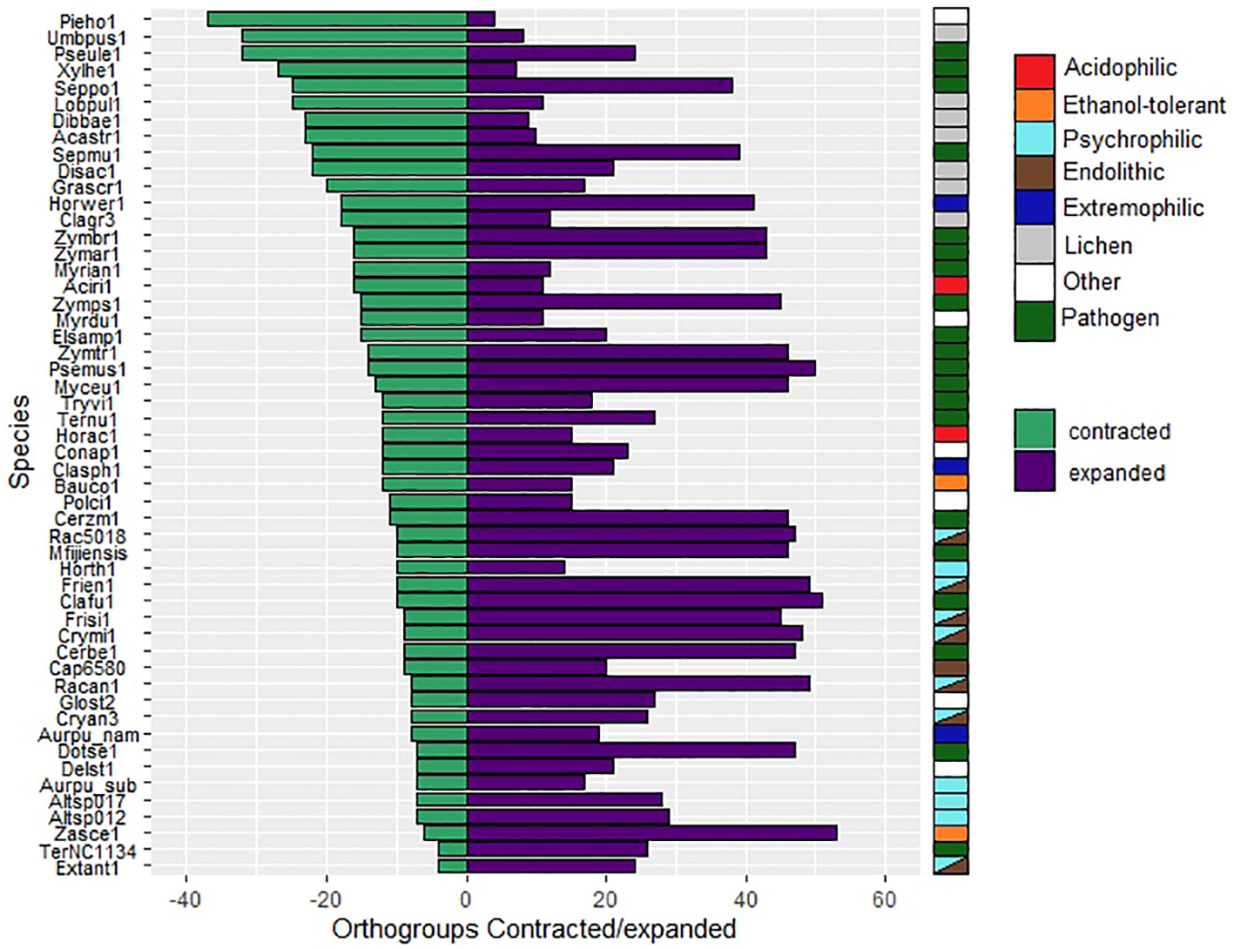
Numbers of orthogroups that are expanded (purple bars) or contracted (green bars) relative to a basal point of zero, no expanded/contracted. Groups are considered to be expanded or contracted in t > abs(0.75). Species are indicated by JGI portal ids (see **Table 1** for full species names).

**Figure 8:**
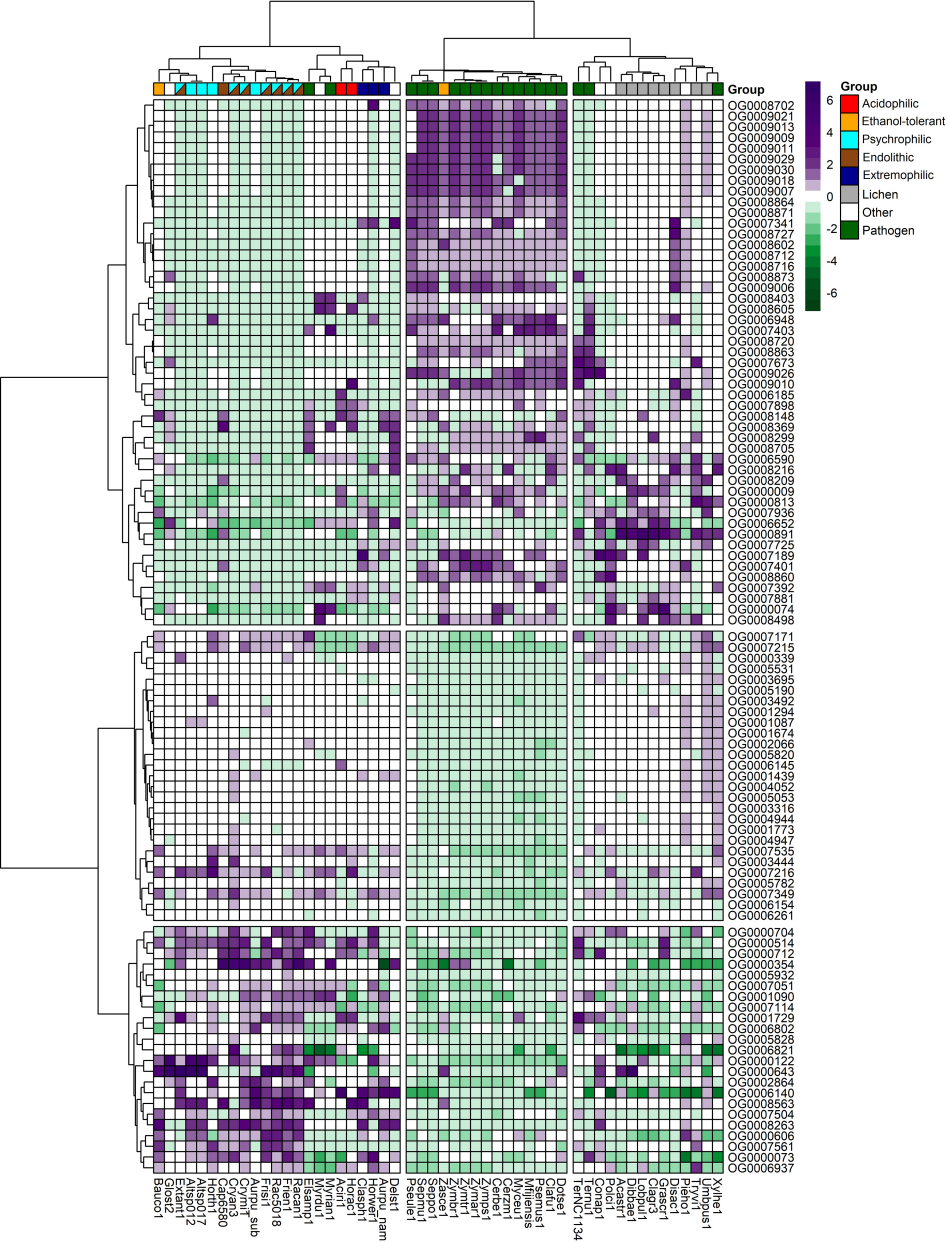
Heatmap of the top expanded and contracted orthogroups in the target psychrophilic species compared to the background fungal species.

The total number of expanded and contracted orthogroups in the fungal species revealed more expansions and fewer contractions in gene families for psychrophilic fungi, while for fungi with other lifestyles, for instance some plant pathogens and lichens, the trend shows more contracted than expanded orthogroups in general (**Figure 7**). The heatmap clustering analysis reveals three distinct groups within the fungal species, which are due to the correlations between expanded and contracted orthogroups across all 52 species (**Figure 8**).

The first group encompasses the psychrophilic species, which notably are clustered with other extremophilic fungi, including the acidophilic, halotolerant, heat tolerant, and the ethanol vapor-tolerant *B. compniacensis*. This implies the existence of a common survival strategy for withstanding environmental conditions associated with cold temperatures among psychrophilic species. These strategies parallel those employed by extremophilic fungi to overcome a diverse array of stresses associated with life under extreme conditions. The heatmap shows the 50 most contracted and expanded orthogroups based on t-score values. There are 30 orthogroups that are expanded in the psychrophilic species but generally contracted in the fungal plant pathogens and lichen species. On the other hand, 49 orthogroups are consistently contracted in the psychrophilic species and expanded in the plant pathogens; while 16 out of these 49 orthogroups are expanded in the third group which encompasses mostly the lichens, along with endophytic, opportunistic pathogens, yeasts and molds (**Figure 8**).

### 3.6 Functional annotation of expanded gene families in psychrophilic species reveals diversification of mechanisms for cold tolerance

We divided the expanded orthogroups into five general categories (**Table 2**) based on their predicted protein functions. We found CAZymes, including glycosyl hydrolases and transferases. Furthermore, enzymes with diverse functions underwent expansion, including carboxylases, peptidases, oxidoreductases, alcohol dehydrogenases, phytanoyl-CoA dioxygenase, and hydrolases. Additionally, we found gene families that function as transporters including at least three orthogroups annotated as major facilitator superfamily (MFS) transporters. Some expanded orthogroups in the psychrophilic species have functional annotations related to DNA and RNA regulation, such as helicases, Zn(2)-C6 transcription factor, G-patch proteins, and Gfd2/YDR514C-like proteins. In addition, a fifth group of annotations consists of stress-related proteins, including cell wall integrity and stress response (WSC) family and aquaporins. Finally, a few orthogroups were uncharacterized or contain domains of unknown function.

**Table 2:** Functional annotations of the top 30 orthogroups that were expanded in psychrophilic species based on t-score analysis.

Orthogroup	Uniprot50 Blast Annotation	Functional group	Number of members
OG0000073	Aquaporin-1		231
OG0000122	Cell wall integrity and stress response component (WSC) domain	Stress-related	185
OG0000354	Gfd2/YDR514C-like C-terminal domain	DNA/RNA regulation	113
OG0000514	Enoyl reductase (ER) domain Alcohol dehydrogenase GroES-like domain	PKS	95
OG0000606	Major facilitator superfamily (MFS) domain	Membrane transporter	87
OG0000643	Clr5 domain*		85
OG0000704	Isochorismatase-like domain		81
OG0000712	NAD(P)-binding protein Cyclin-dependent kinases regulatory subunit		80
OG0001090	3-phytase Phosphoglycerate mutase-like protein		65
OG0001729	MFS-type transporter prx5 Major facilitator superfamily (MFS) profile domain	Membrane transporter	60
OG0002864	MFS general substrate transporter Major facilitator superfamily (MFS) profile domain	Membrane transporter	58
OG0003444	Chromo domain		57
OG0005782	BTB domain		52
OG0005828	Zn(2)-C6 fungal-type domain	DNA/RNA regulation	52
OG0005932	Pyruvate carboxylase		52
OG0006140	AB hydrolase-1 domain		50
OG0006802	Glycoside hydrolase family 1 protein	CAZyme	42
OG0006821	Uncharacterized protein		42
OG0006937	EthD domain	Oxidoreductase	40
OG0007051	Phytanoyl-CoA dioxygenase family protein	Stress-related	38
OG0007114	Uncharacterized protein		37
OG0007171	MFS general substrate transporter	Membrane transporter	36
OG0007215	G-patch domain	DNA/RNA regulation	35
OG0007216	Dienelactone hydrolase domain		35
OG0007349	Oxidoreductase		32
OG0007504	Peptidase M20 dimerization domain		30
OG0007535	Helicase-associated domain Helicase-like protein	DNA/RNA regulation	29
OG0007561	Alpha-mannosyltransferase alg11p protein		29
OG0008263	Succinate dehydrogenase assembly factor 4, mitochondrial	PKS	20
OG0008563	Zn(2)-C6 fungal-type domain	DNA/RNA regulation	17

*Only the consensus representative sequence of the Uniref50 cluster obtained by BLAST supported the annotation but no other members in the cluster have a reported annotation.

### 3.7 Gene families contracted in psychrophilic fungi are mostly required for interaction with plants and microorganisms

Annotation analysis of the gene families that were contracted in psychrophilic fungi revealed that the same orthogroups were often expanded in both psychrophiles (section 3.6) and plant pathogens. Our analysis identified a diverse array of annotations, including proteins involved in pathogenicity, stress response, effector production, cell wall integrity, and production of secondary metabolites (**Table 3**). Interestingly, the Ecp2 effector gene family, which is widely distributed among Dothideomycetes and involved in host-pathogen interactions, is expanded in the plant pathogens but contracted in psychrophiles. Several orthogroups are associated with cell wall integrity, featuring annotations such as cell wall proteins, hydrophobic surface-binding proteins, and acetyltransferases. In terms of stress response, we found gene families annotated as methyltransferases, and NACHT domain-containing proteins. Furthermore, multiple gene families associated with pathogenicity were identified, including F-box proteins, extracellular membrane proteins, oxidases, and enzymes for cell wall degradation and nutrient uptake. A remarkable finding was the expansion of the Zn(2)-C6 fungal-type domain, which can act as an aflatoxin regulatory protein according to its Interpro annotation.

**Table 3:** Functional annotations of the top 49 orthogroups that were contracted in psychrophilic species based on t-score analysis.

Orthogroup	Uniprot50 Blast Annotation	Functional group	Number of members
OG0008702	FAD dependent oxidoreductase domain		16
OG0009021	NACHT-NTPase and P-loop NTPases N-terminal domain	Stress response – microbiome interaction	14
OG0009013	Fungal specific transcription factor domain	DNA/RNA regulation	14
OG0009009	Chromo domain-containing protein*		14
OG0009011	Uncharacterized protein		14
OG0009029	KTSC domain-containing protein*		14
OG0009030	Extracellular membrane protein CFEM domain-containing protein	Pathogenicity/Interaction with environment	14
OG0009018	Ca2+-modulated nonselective cation channel polycystin*		14
OG0009007	Uncharacterized protein		14
OG0008864	Uncharacterized protein		15
OG0008871	Fungal-type protein kinase domain		15
OG0007341	AA1-like domain	Enzymes for degradation	33
OG0008727	BZIP domain 2-hydroxyphytanoyl lyase	Transcription Factor. DNA/RNA regulation	16
OG0008602	C2H2-type domain	DNA/RNA regulation	17
OG0008712	BZIP transcription factor like protein	Transcription Factors and Regulatory Proteins	16
OG0008716	Uncharacterized protein		16
OG0008873	3-phytase		15
OG0009006	Uncharacterized protein		14
OG0008403	Pyrroline-5-carboxylate reductase	Stress-response	19
OG0008605	cellulose synthase (UDP-forming)		17
OG0006948	Hydrophobic surface binding protein	Cell wall integrity	40
OG0007403	Ecp2 effector-like protein	Pathogenicity	32
OG0008720	Peptidase M24 domain	Pathogenicity	16
OG0008863	Fumarylacetoacetase-like C-terminal domain 2-hydroxyhexa-2,4-dienoate hydratase	Pathogenicity	15
OG0007673	F-box domain	Pathogenicity	28
OG0009026	ATP-grasp fold PylC-type domain		14
OG0009010	Uncharacterized protein		14
OG0006185	DUF4185 domain		50
OG0007898	Uncharacterized protein		24
OG0008148	C2H2-type domain	DNA/RNA regulation	21
OG0008369	N-acetyltransferase domain	Cell-wall integrity	19
OG0008299	Manganese lipoxygenase	Enzyme	20
OG0008705	NAD(+) diphosphatase		16
OG0006590	Zn(2)-C6 fungal-type domain	DNA/RNA regulation	45
OG0008216	Ubiquitin-like protease family profile domain		20
OG0008209	NWD NACHT-NTPase N-terminal domain	Stress response – microbiome interaction	20
OG0000009	Tat (Twin-arginine translocation) pathway signal sequence		526
OG0000813	Multicopper oxidase	Pathogenicity	75
OG0007936	CCHC-type domain	DNA/RNA regulation	24
OG0006652	NACHT domain	Stress response – microbiome interaction	44
OG0000891	Major facilitator superfamily (MFS) profile domain	Transporter	71
OG0007725	Pectin lyase-like protein	Enzyme/plant cell wall degradation	27
OG0007189	Uncharacterized protein		35
OG0007401	AA1-like domain	Enzymes for degradation	32
OG0008860	MATE efflux family protein	Transporter	15
OG0007392	AB hydrolase-1 domain		32
OG0007881	GPR1/FUN34/YaaH-class plasma membrane protein	Membrane receptor, pathogenicity	25
OG0000074	Nonribosomal peptide synthetase TES	Pathogenicity	230
OG0008498	Methanethiol oxidase		18

*Only the consensus representative sequence of the Uniref50 cluster obtained by BLAST supported the annotation but no other members in the cluster have a reported annotation.

## 4 Discussion

Previous analyses did not reveal much that was unique in the draft genome of a different isolate of *C. antarcticus*, and concluded with the question, “Nothing special in the specialist?” (Sterflinger et al., 2014). The previously identified haploid genome size and total GC contents were similar to our results. However, that genome was generated with short-read technology that yielded a very fragmented assembly containing over 12,400 contigs, compared to our PacBio assembly of isolate CBS 116301 of only 262 contigs. By greatly increasing the number of genomes from fungi with different lifestyles for comparative analyses it was possible to identify systematically expanded or contracted gene families in *C. antarcticus* and other psychrophiles that provide clues about their adaptations to extreme cold.

An unexpected result from our analysis is that many of the smaller contigs appear to represent an alternative haplotype to the major contigs, indicating that the sequenced isolate was likely diploid or dikaryotic. This is surprising because these fungi are believed to be haploid and asexual (Nieuwenhuis and James, 2016; Selbmann et al., 2020). The presence of an alternate “haplotigs” set has been reported in phased diploid genome assemblies of plant pathogenic strains of *Candida albicans* (Hamlin et al., 2019). In this species, considered a strict diploid, the genome assembly is composed of two sets of contigs; a primary contigs set, and an alternate haplotigs set. The latter one represents alternate haplotypes in the regions for which phasing was achieved for the genome assembly. The N50 metric for the primary contigs in *C. albicans* approached 2 million bp, indicating substantial contiguity. In contrast, the N50 values for the haplotigs ranged from 170,000 bp to 254,000 bp, indicating that contigs representing an alternative haplotype are shorter in length (Hamlin et al., 2019), as was also seen in *Cryomyces antarcticus*. Additional evidence of diploidy in *C. antarcticus* was obtained through a k-mer frequency plot, which displayed two major peaks of different heights (**Supplementary Figure 2**). This bimodal distribution is characteristic of a diploid genome (Ranallo-Benavidez et al., 2020). Similar findings have been observed in the entomopathogenic fungal genus *Metarhizium*, typically a haploid organism, with an instance of diploidy described in the species *M. acridum* (Nielsen et al., 2021).

Whether the expanded genome of *C. antarcticus* represents a stage in the process of sexual reproduction, a balanced heterokaryon, or some other phenomenon such as whole-genome duplication is not known and should be the subject of additional research. Future experiments might include fluorescence flow cytometry analysis on multiple isolates of *C. antarcticus*. This analysis would determine whether some isolates contain twice the cellular DNA compared to a haploid control strain or a closely related haploid fungal organism (Lin et al., 2009). Additionally, 4,6-diamidino-2-phenylindole (DAPI) nuclear staining using fluorescence microscopy could be applied to test for cells with a single nucleus, confirming diploidy. Alternatively, identifying two independent haploid nuclei would indicate dikaryosis (Lin et al., 2009). The DAPI technique has traditionally been used as a rapid method to determine ploidy in fungal cells and was first assessed in the model yeast *Schizosaccharomyces pombe* (Talbot et al., 1988). A further way to test for ploidy would be to assess the genome composition of single-ascospore isolates. If single-ascospore isolates have half the DNA content and show no evidence of duplicated sequences then the original isolate was likely diploid. If they have the same genomic composition that would support the hypothesis of a whole-genome, autopolyploid duplication or possibly an allopolyploidization event from hybridization with a closely related species. However, these experiments would depend on generating the sexual stage, which so far remains unknown.

Due to differences in sequencing technologies caution should be applied when comparing contig-level genome assemblies, particularly when assessing features of genome evolution such as RIP, GC contents, and genome size. We observed a significant genome size difference between *C. minteri* and *C. antarcticus*, which is larger than expected given their close phylogenetic relationship, but may be explained by differences in sequencing and assembly methods, ploidy, and repeat content. Pepijn and Pellicer (2020) showed that genome assembly size depends on the software used. In that analysis, the genome of two isolates of *Leucocoprinus* sp. and one of *Leucoagaricus barssii* were assembled using four software packages (ABySS 2.0.0, SGA 0.10.15, SPAdes 3.7.1 and SOAPdenovo 2.04) showing considerable differences in the total lengths of the assemblies, ranging from 41 to 172 Mb for the same isolate. A similar difference in size was observed between the genomes of the closely related pathogens *Epichloe typhina* and *E. clarkia*, where size discrepancies have been attributed to the impact of RIP on genome expansion (Treindl et al., 2021). Repeat-Induced Point mutation (RIP) functions as a defense mechanism in fungal genomes that counteracts the activity of transposable elements to minimize their proliferation (Gladyshev, 2017). This defense strategy induces C-to-T transition mutations within duplicated DNA sequences throughout the genome, resulting in the formation of GC-depleted regions and AT-rich sequences (Hood et al., 2005; Testa et al., 2016). RIP occurs during the sexual stage in haploid nuclei after fertilization but prior to meiotic DNA replication (de Wit et al., 2012). Our analysis revealed traces of RIP in the *C. minteri* and *C. antarcticus* genomes, covering roughly 4% of each genome. While the genome-wide RIP percentages observed in both *Cryomyces* species do not rank among the highest reported for fungi in the class Dothideomycetes (Nagel et al., 2021), they do represent the highest within the psychrophilic fungi in our analysis and indicate that these species must undergo sexual reproduction at least occasionally, since RIP acts only during the sexual cycle (Galagan and Selker, 2004).

In our analysis, we did not investigate the presence of sexual mating type genes. However, a previous study of the *C. antarcticus* genome by Sterflinger et al. (2014) identified 32 mating genes and 34 mating proteins using BlastP searches of the annotated proteins against all available mating proteins in the GenBank database of fungal species. The homologs of these mating genes were identified in the classes Eurotiomycetes, Sordariomycetes, and Dothideomycetes (Sterflinger et al., 2014). This finding, along with the likely diploidy of *C. antarcticus*, suggests its potential capability for sexual reproduction. However, to confirm a sexual cycle in this fungus, the functionality of the mating type genes needs further investigation (Usher, 2019). Additionally, we observed a maximum heterozygosity level of 1.15% in *C. antarcticus* based on the kmer frequency analysis, indicating some degree of genetic recombination and, consequently, possible sexual reproduction assuming the fungus is diploid. If it is not a diploid, then the low level of heterozygosity supports whole-genome duplication by an autopolyploidization event rather than allopolyploidy resulting from hybridization. Despite the evidence of a possible sexual stage, the teleomorph of *C. antarcticus* remains undiscovered under laboratory conditions. This absence of experimental evidence might be due to the silencing of mating genes or the presence of cryptic sexual cycles triggered by specific environmental conditions, or infrequent sexual reproduction events (Sterflinger et al., 2014; Usher, 2019).

Research on RIP and TE content in psychrophilic and extremophilic fungi is scarce. One study, focusing on the psychrophilic fungus *M. psychrophile*, revealed minimal repeat sequence content (Su et al., 2016). Currently, most of the research on RIP and TEs predominantly centers on plant pathogens. In fact, among the species included in our analysis, plant pathogens exhibited the highest RIP percentages; this was especially evident in the pathogens *P. ulei, P. fijiensis, P. musae, C. fulvum* and *P. eumusae*, which all show more than 35% RIP. A high percentage of RIP has been reported previously in other fungal plant pathogens in the Dothideomycetes (de Wit et al., 2012; Ohm et al., 2012; Isaza et al., 2016).

While research on extremophiles in this area is limited, the impact of RIP has been extensively explored in Dothideomycetes plant pathogens including *Z. tritici* and its sister species, where a significant proportion of TEs exhibit RIP-like signatures (Lorrain et al., 2021). Analyses of *Z. tritici* isolates have revealed a higher incidence of RIP and TEs in accessory chromosomes compared to core chromosomes (Badet et al., 2020; Lorrain et al., 2021). Notably, *Z. tritici* maintains most of its effector genes on accessory chromosomes, often adjacent to TEs, indicating the crucial role of TEs in shaping the evolution of pathogenicity genes in fungi (Lorrain et al., 2021). Drawing parallels with plant pathogens, where key virulence genes are subject to RIP-induced changes, it is conceivable that RIP serves as a driver of gene evolution in extremophilic fungi, facilitating adaptation to extreme conditions. Given the necessity for environmental adaptation in extremophiles, similar to the importance of virulence in plant pathogens, RIP may contribute significantly to the evolutionary dynamics of genes involved in survival mechanisms. However, this hypothesis needs to be evaluated with a deeper analysis of large population-genomics datasets of *Cryomyces* species to test for RIP and where it occurs relative to chromosomal position.

GC content provides a simple overall indicator of nucleotide composition and can reveal variations in genomic stability and resistance to environmental stressors (Testa et al., 2016; Hu et al., 2022). At low temperatures, base-paired DNA and RNA structures are stabilized. To allow essential processes like replication and transcription to continue normally, one might expect to see lower GC content in psychrophiles. Surprisingly, we observed higher GC contents in the genomes, CDS and GC3s of the psychrophilic fungi compared to mesophiles. A Tukey’s test revealed statistically significant differences in genome-wide GC content between psychrophiles and two major comparison groups: plant pathogens and lichens. This suggests that the GC content of the genome might be a distinctive feature of psychrophilic fungi. Previous analyses reported similar results. For instance, the GC contents in psychrophilic yeasts are higher than they are in those that are mesophilic (Liu et al., 2023). In the fungus *Mrakia psychrophila*, overall GC content surpassed that of mesophilic fungi such as *C. neoformans*. This result extended to the GC contents of coding sequences (CDS), which were 56.5% and 56.8% in the two psychrophilic species *M. psychrophila* and *D. cryoxerica*, respectively. A similar pattern, with even more pronounced differences, was observed for the third bases of codons (GC3) in both *M. psychrophila* and *D. cryoxerica*, compared to *C. neoformans* (Su et al., 2016).

GC base pairs in DNA are more stable than AT base pairs (See Ghosh et al., 2020a and Mo, 2006). This is not due to hydrogen bonding, which contributes minimally to DNA stability, but rather to Van der Waals and electronic interactions between the stacked base-pairs in the DNA helix (Ghosh et al., 2020a). Entropic effects play a huge role in DNA helix stability (which is why DNA denatures at high temperatures), with the result that DNA helices are more stable at low temperatures. What then could cause the observed increase in GC content in psychrophiles? Recent results show that solvent viscosity decreases helix stability, possibly due to molecular crowding effects (Ghosh et al., 2020b; Stellwagen and Stellwagen, 2019). High viscosity also destabilizes initiation of RNA stem formation and increases the destabilizing effect of terminal AU base-pairs (Ghosh et al., 2020b). Fungal psychrophiles produce cryoprotectants such as glycine betaine, trehalose, glycerol, sucrose, sarcosin, mannitol, sorbitol, 2-polyalkanoates, and exopolysaccharides (Chen et al., 2022; Pacheco et al., 2019; Ghobakhlou et al., 2015; Goordial et al., 2016; Su et al., 2016; Deegenaars and Watson, 1997). Such cryoprotectants depress the freezing point and decrease water loss, both important to survival in the cold, dry Antarctic environment. These compounds probably also increase the viscosity of the intracellular environment, leading to DNA helix destabilization, and destabilization of RNA helix initiation. This may provide an explanation for the otherwise non-intuitive increase in genome-wide GC contents in psychrophiles.

Expansions and contractions of gene families may provide additional clues about adaptations to an extreme environment. For example, in the psychrophilic species we found expansions of helicases and G-patch domain-containing proteins, which are involved in multiple processes for genome regulation. In other organisms, DEAD-box RNA helicases are involved in regulation of nucleic acid binding, modulation of RNA/DNA: DNA interactions, and RNA metabolism, including translation initiation and ribosome biogenesis due to their capability to unwind the duplex RNA in eukaryotes (Lim et al., 2000; Taschuk and Cherry, 2020). In psychrophilic microorganisms RNA helicases are differentially expressed at low temperatures (Feller, 2013) and they can act as nucleic acid chaperones that help the microorganisms adapt to cold conditions (Xu et al., 2024). In particular, DEAD-box RNA helicases can presumably enable some bacteria to survive cold shock and grow at low temperatures, due to their role in removing cold-stabilized secondary structures in mRNA (Lim et al., 2000). In the Antarctic archaeon *M. burtonii*, an RNA helicase gene was highly transcribed during cell growth at 4°C, revealing the importance of these proteins for low-temperature adaptation (Lim et al., 2000). G-patch proteins are involved in regulation and activation of helicases. The first characterized protein with a G-patch domain that regulates an RNA helicase was Spp2 (Roy et al., 1995). The *in vivo* interaction of Spp2 was first documented in budding yeast, where a DEAH/RHA helicase named Prp2 interacts physically with Spp2, and this interaction is required for the activation of Prp2 function. Also, a helicase (Prp43) that is required in both splicing and ribosome biogenesis, is activated by a G-patch protein (Ntr1) which forms a stable complex with another G-patch protein (Ntr2). This complex recruits the helicase Prp43 targeting its helicase activity for spliceosome dissociation (Robert-Paganin et al., 2015).

Another gene family that was expanded in the psychrophiles was Zn(2)-C6 transcription factors, which are exclusively found in fungi. They can act as repressors, activators, or both, depending on the target genes (MacPherson et al., 2006). The regulatory scope of these transcription factors is broad, including activation of sterol biosynthesis (Vik and Rine, 2001), modulation of multidrug sensitivity (Coste et al., 2004), and regulation of genes involved in galactose metabolism (Breunig, 2000). Previous analyses have highlighted the versatility of Zn(2)-C6 transcription factors. It is particularly interesting that, in *Colletotrichum lagenarium*, Cmr1p positively regulates the expression of genes involved in melanin biosynthesis, which is crucial for successful invasion of host plants during anthracnose of cucumbers (Tsuji et al., 2000). In rock-inhabiting psychrophilic fungi, melanin biosynthesis is crucial for survival because melanin pigments protect the cells from extreme temperatures, solar irradiation, extreme osmotic and pH conditions, and provide tolerance to toxic metals (Selbmann et al., 2015). Moreover, Stb5, a Zn(2)-C6 transcription factor in *Saccharomyces cerevisiae*, can switch between activator and repressor roles under oxidative stress conditions (Larochelle et al., 2006). It binds to and regulates the expression of genes in the pentose phosphate pathway and those involved in NADPH production, which is essential for oxidative stress resistance. In fact, the activation of mechanisms for protection against oxidative stress plays a crucial role in psychrophilic fungi. It has been shown that a cold environment induces the production of reactive oxygen species (ROS), which can cause severe damage to DNA, lipids and proteins, affecting survival of the cell (Kostadinova et al., 2017). The expansion of genes for Zn(2)-C6 transcription factors in *C. antarcticus* and other psychrophilic extremophiles may help to counter this damage.

Another expanded group includes major facilitator superfamily (MFS) transporters which likely are involved in drug resistance and amino acid uptake. The MFS are secondary transporters that have primary roles in antifungal resistance and nutrition transport. They can indirectly control membrane potential by changing membrane lipid homeostasis, and regulate internal pH and the stress-response machinery in fungi (Su et al., 2016; Chen et al., 2017). MFS transporters are expanded in extremophilic fungal species such as *Mrakia psychrophila* and *Penicillium funiculosum* where they are required for accumulation of nutrients from the environment and resistance to acidic stress, respectively (Xu et al., 2014; Su et al., 2016). In *Alternaria alternata*, an MFS transporter named AaMFS19 is required for cellular resistance to oxidative stress, including H_2_O_2_, KO_2_, and other oxygen-generating compounds (Chen et al., 2017).

Some enzymes play a role in alternative pathways for energy production in fungi. We observed expansion of gene families encoding alcohol dehydrogenases. The expression of this family of enzymes has been reported, along with increased expression of glycolytic genes that feed into ethanol production via fermentation, in the Antarctic yeast *Rodothorula frigidialcoholis* growing at 0°C (Touchette et al., 2022). This suggests that *R. frigidialcoholis* adapts to cold temperatures by redirecting pentose phosphate pathway molecules to ethanol fermentation. This switch from respiration to fermentative metabolism to produce energy contributes to its survival in extreme cryoenvironments (Touchette et al., 2022). In *Mucor lusitanicus*, a knock-out strain of the alcohol dehydrogenase gene (*adh1*), showed reduction in lipid and fatty acid content, which was restored by supplementing the medium with external ethanol, suggesting that alcohol dehydrogenases are also involved in lipid biosynthesis (Gutiérrez-Corona et al., 2023). The expansion of genes for alcohol dehydrogenases in psychrophiles might be involved either in the activation of alternative pathways for energy production, or in specific stages of lipid biosynthesis, both of which could be alternative mechanisms of adaptation to life in extremely cold environments.

The expansion of CAZymes in the psychrophilic species may facilitate cell wall modifications and energy storage. Fungi living in cold environments maintain cell integrity by remodeling the cell wall. CAZymes control the repair and modification of cell wall components necessary to overcome extreme low temperatures and desiccation. For example, in *Candida albicans* the architecture of the cell wall undergoes alterations in response to environmental conditions, resulting in changes to its composition and structure in newly synthesized cells (Ene et al., 2015). In previous analyses, extremotolerant species have been shown to encode more members of the CAZyme family glycosyl hydrolase 32 (GH32) compared to opportunistic, non-opportunistic, and pathogenic species (Gostinčar et al., 2018). This family of CAZymes contains invertases and other enzymes involved in energy storage and recovery. In addition, studies have concluded that fungal cell wall elasticity is critical for survival under conditions of osmotic shock (Ene et al., 2015). Structural changes that improve cell wall elasticity involve cross-linking between cell wall macromolecules, which is catalyzed by the action of carbohydrate-active cell wall-remodeling enzymes, including hydrolases, transglycosidases, and transferases. These include the Gas-like family of β-1,3-glucanosyltransferases, and a family of chitin-glucanosyltransferases, which encompasses family GH16 (Ene et al., 2015; Ibe and Munro, 2021). In the fungal pathogen *B. cinerea*, the GT2 family contributes to the biogenesis and remodeling of the cell wall. This family contains the synthases for production of chitin, which plays a structural role in the fungal cell wall (Blandenet et al., 2022). In our analysis, we observed expansion of gene families of AB hydrolases (OG0006140), glycoside hydrolase family 1 (OG0006802), and the alpha-mannosyltransferase protein (OG0007561), which may be involved in cell wall modifications in response to cold temperatures.

Other expanded families, such as the phytanoyl-CoA dioxygenase proteins, are peroxisomal enzymes that catalyze the first step of phytanic acid alpha-oxidation. In *Streptomyces coelicolor*, an ectoine dioxygenase is involved in the synthesis of 5-hydroxyectoine, a compatible solute that is synthesized upon exposure to high salinity (Bursy et al., 2008). This compound helps organisms to survive extreme osmotic stress by acting as a highly soluble organic osmolyte. Bursy et al. (2008) also found that synthesis of 5-hydroxyectoine by *S. coelicolor* is a stress response that protects against conditions of high salt and heat.

In contrast to gene families that were expanded in the psychrophiles, others were reduced, especially in comparison to the plant pathogens, in which gene families involved in microbiome interactions and defense or immune responses often were expanded. These families were contracted in psychrophilic fungi, most likely because plant pathogens require more proteins associated with microorganism and plant interactions, or interactions with other living things in general, while psychrophilic fungi are more isolated and face bigger challenges from abiotic and environmental factors rather than from other living organisms.

Most of the orthogroups that are contracted in the psychrophiles are proteins related to signaling and the membrane. They show diverse functions from degradation, response to stress, and synthesis of new compounds. Acetyltransferase (GNAT), heme haloperoxidase, and peroxidase are common domains in the contracted orthogroups. These proteins are involved in lignin and chitin degradation, which mostly are not used by cold-tolerant fungi (Leung et al., 2011). However, the insect pathogen *Lecanicillium muscarium*, which was isolated from Antarctica, is a high producer of cold-tolerant enzymes, including those for hydrolyzing chitin, as expected for a fungus that feeds on arthropods (Fenice, 2016). Several fungi isolated from King George Island, Antarctica, including the Dothideomycetes *Penidiella kurandae*, showed amylase and cellulase activities, even when grown at 4°C (Krishnan et al., 2016).

Other reduced orthogroups in the psychrophilic fungi are associated with virulence/pathogenicity and toxin production in plant pathogens such as polyketide synthase, glycoside hydrolase, ribonuclease ribotoxin, aflatoxin regulatory protein, and pyrroline-5-carboxylate reductase. A previous comparative genomic analysis of 20 dothideomycetous and eurotiomycetous black fungi showed a lack of specialized virulence traits in extremotolerant and opportunistic fungi (Gostinčar et al., 2018). This is consistent with our observed contraction of these orthogroups.

We observed expansion of the NACHT-NTPase and P-loop NTPase N-terminal domain gene families in the group of plant pathogens, which suggests involvement of these proteins in controlling a variety of biotic interactions, not strictly limited to immune responses. The nucleotide-binding oligomerization domain (NOD)-like receptors (NLRs) are signal-transducing proteins that control innate immunity in plants, animals and fungi (Dyrka et al., 2014). In fungi, NACHT domain proteins, a subtype of NLRs, control immunity and other nonpathogenic biotic interactions, such as symbiotic relationships with microbiomes (Chu and Mazmanian, 2013). The HET-E protein of *Podospora anserina*, which first defined the NACHT domain, is involved in fungal non-self recognition and programmed cell death due to heterokaryon incompatibility (Saupe et al., 1995; Koonin and Aravind, 2000). Additionally, studies have shown that NACHT domain proteins are specifically expressed during mycorrhizal symbiosis in certain fungi (Martin et al., 2008). These interactions could include fungal pathogenicity and symbiotic interactions (such as ECM formation, endophytic growth, lichen formation, or microbiome interactions).

In support of this idea, some studies suggest that variation in the number of NLR homologs among different fungal species might be associated with the diversity of ecological niches and lifestyles (Dyrka et al., 2014). For example, highly versatile pathogens like *Fusarium* species or mycoparasitic *Trichoderma* species tend to have large NLR repertoires, possibly for protection against microbial competitors. However, the ethanol-tolerant *B. compniacensis* only has a small repertoire of these NLRs, which might result from inhabiting a restrictive niche (Dyrka et al., 2014). Similarly, our results suggest that extremophilic fungi which inhabit environments with a restricted number of microbial competitors and potential hosts, might not require specialized mechanisms for biotic interactions, which explains the contraction of the NACHT domain-containing protein family.

The contraction of hydrophobic surface-binding proteins among the psychrophiles may be due to the involvement of hydrophobins in water-sensing mechanisms during spore germination. Hydrophobins contribute to the surface hydrophobicity needed for conidial dispersal, and protection against a host defense system (Tanaka et al., 2022), all of which are essential processes for pathogenic or symbiotic interactions but are not required in non-pathogenic, psychrophilic fungi. In plant pathogens like *M. grisea* and *F. graminearum*, hydrophobins are required for production of infective structures, penetration of the water-air interface of the host and attachment to hydrophobic surfaces (Talbot et al., 1993; Quarantin et al., 2019). These properties are not required by saprobic, extremophilic psychrophiles and this is reflected in their proteome complements.

In addition to the gene families discussed above, we found that genes involved in stress responses against UV irradiation, oxidative stress, and antifungal agents, are also contracted. This finding is remarkable given that the Antarctic continent receives the highest levels of UV radiation on Earth. In 2020, the continent experienced the highest UV irradiances in more than two decades, a consequence of the recurring Antarctic ozone hole that appears every spring (Cordero et al., 2022). The endolithic lifestyle of most psychrophilic fungi in this study might explain the contraction of gene families involved in stress responses to UV radiation. The lithic environment provides a refuge against harsh weather conditions such as high UV radiation and water scarcity. For example, expansion of hyphal growth into deeper rock layers helps to protect the cells of rock-inhabiting fungi from UV radiation (Gorbushina, 2007). Consequently, psychrophiles living as endolithics can withstand severe radiation levels and other stress forms. Indeed, endolithic communities consist of some of the most extremophilic and resilient microorganisms known to date (Coleine et al., 2021). These microorganisms migrate into fractures or in pore spaces where the necessary nutrient, moisture, and light levels are sufficient for survival (Stivaletta and Barbieri, 2008). Thus, the endolithic lifestyle not only provides protection against radiation but also serves as a strategy to avoid water stress and access a more humid environment in some of the driest and coldest places on Earth. A future analysis that includes a broader range of extremophilic fungal species with both epilithic and endolithic lifestyles is of interest to determine the differences in gene families that are expanded and contracted in endolithics compared to epilithics. We likely would not observe the contraction of gene families associated with UV stress response in a group predominantly composed of epilithic or epiphytic psychrophiles. This finding suggests that evolution and adaptation may have more to do with a dynamic and diversified set of strategies, as opposed to using only a specific set of proteins and mechanisms to cope with extreme environments.

While at first glance there was nothing special in the specialist (Sterflinger et al., 2014), upon a closer look with a larger cohort of fungi plus a much-improved genome sequence, expanded and contracted gene families could be identified and provide clues about likely adaptations to extreme conditions. This analysis highlights the importance of a large number of genomes available for comparison and validates the goals of programs like the JGI 1000 Fungal Genomes project (Grigoriev et al., 2014) which seeks to generate the requisite sequences. In a sense, Sterflinger et al. (2014) were correct in that there were not radical changes in gene composition that distinguish psychrophiles from other fungi. Instead, the changes most likely reflect a slow adaptation of *C. antarcticus* and its relatives over long periods of evolutionary time as gene families expanded or contracted and homologs changed function as they were selected for increasing tolerance to cold stress. Clearly much additional work is needed to understand the expression of the identified gene families under extreme conditions to fully elucidate the ability of *Cryomyces* to grow under conditions that are more similar to those found on Mars (Onofri et al., 2004; 2015) than they are to most areas of Earth.

## Data Availability

The datasets presented in this study can be found in online repositories. This Whole Genome Shotgun project has been deposited at DDBJ/ENA/GenBank under the accession JBDRTV000000000. The version described in this paper is version JBDRTV010000000.

## Supplementary material

Supplementary Figure 1 VISTA dot plot of *Cryomyces antarcticus* contigs against themselves based on the whole-genome DNA alignment (Dubchak, 2007). The red and blue diagonal lines are nucleotide matches between sequences on different contigs, indicating DNA duplication. The main diagonal of self alignment has been removed for clarity. A higher quality version of the supplementary figure can be accessed at https://mycocosm.jgi.doe.gov/vista_embed/?viewMode=dotPlot&organism=Cryan3&?&run=8474-crZbaJ90&xdset=3156&ydset=3714&cutoff=1000

Supplementary Figure 2 K-mer frequency plot of the circular consensus sequencing data from *Cryomyces antarcticus* using a k-mer length of 31 nucleotides. Two peaks indicating diploidy or dikaryosis are shown: a taller peak with high-frequency k-mers and a smaller peak with low-frequency k-mers that are unique to one heterologous chromosome. The other small peaks possibly indicate repeats in the genome.

Supplementary Table 1 Repeat-induced point mutation (RIP) in the genomes used for comparative analysis.
