# Supplementary material for: Comparative genomics of the extremophile Cryomyces antarcticus and other psychrophilic Dothideomycetes
Source: Front Fungal Biol. 2024 Sep 6;5:1418145. doi: 10.3389/ffunb.2024.1418145 (PMC11412873; doi:10.3389/ffunb.2024.1418145)
Supplement: Supplementary Figure 1 — VISTA dot plot of Cryomyces antarcticus contigs against themselves based on the whole-genome DNA alignment (Dubchak, 2007). The red and blue diagonal lines are nucleotide matches between sequences on different contigs, indicating DNA duplication. The main diagonal of self alignment has been removed for clarity. A higher quality version of the supplementary figure can be accessed at https://mycocosm.jgi.doe.gov/vista_embed/?viewMode=dotPlot&organism=Cryan3&?&run=8474-crZbaJ90&xdset=3156&ydset=3714&cutoff=1000 [file DataSheet1.docx]

Supplementary table 1. Repeat-induced point mutation (RIP) in the genomes used for comparative analysis.

| **Species name** | **JGI Portal ID** | **Total estimated genome wide RIP (%)** | **Average size of LRAR (kbp)** | **Average GC content of LRAR (%)** | **Sum of all LRAR (Mbp)** | **Composite index for LRARs** |
| --- | --- | --- | --- | --- | --- | --- |
| *Acarospora strigata* | Acastr1 | 0.09 | 0 | 0 | 0 | 0 |
| *Acidomyces richmondensis BFW* | Aciri1 | 3.2 | 6.1 | 40.4 | 0.2 | 1.05 |
| *Aureobasidium namibiae* | Aurp_nam1 | 0.77 | 7.38 | 37.27 | 0.1 | 1.32 |
| *Aureobasidium subglaciare* | Aurpu_sub1 | 1.18 | 9.17 | 37.41 | 0.1 | 1.66 |
| *Baudoinia compniacensis* | Bauco1 | 0.78 | 8.98 | 32.43 | 0.1 | 1.57 |
| *Cercospora berteroae* | Cerbe1 | 4.75 | 7.27 | 40.6 | 0.2 | 1.32 |
| *Cercospora zeae-maydis* | Cerzm1 | 29.67 | 13.32 | 36.75 | 9.2 | 1.73 |
| *Cladosporium fulvum* | Clafu1 | 42.2 | 10.07 | 42.18 | 10.3 | 1.73 |
| *Cladonia grayi* | Clagr3 | 12.61 | 12.72 | 15.8 | 2.4 | 1.11 |
| *Cladosporium sphaerospermum UM 843* | Clasph1 | 0 | 0 | 0 | 0 | 0 |
| *Coniosporium apollinis* | Conap1 | 9.65 | 14.96 | 30.04 | 1.9 | 1.45 |
| *Cryomyces antarcticus* | Cryan3 | 4.04 | 10.97 | 39.27 | 1.5 | 1.58 |
| *Cryomyces minteri CCFEE 5187* | Crymi1 | 4.36 | 9.42 | 37.36 | 1 | 1.55 |
| *Delphinella strobiligena* | Delst1 | 5.56 | 5.83 | 23.08 | 0.9 | 0.85 |
| *Dibaeis baeomyces* | Dibbae1 | 0.21 | 0 | 0 | 0 | 0 |
| *Dissoconium aciculare* | Disac1 | 0.75 | 8.58 | 26.04 | 0.1 | 1.3 |
| *Dothistroma septosporum* | Dotse1 | 3.66 | 30.74 | 31.59 | 1.1 | 1.7 |
| *Elsinoë ampelina* | Elsamp1 | 17.82 | 18.16 | 31.11 | 4.3 | 1.69 |
| *Friedmanniomyces endolithicus CCFEE 5311* | Frien1 | 2.4 | 9.04 | 42.57 | 0.7 | 1.52 |
| *Friedmanniomyces simplex CCFEE 5184* | Frisi1 | 3.62 | 7.15 | 41.68 | 0.3 | 1.43 |
| *Glonium stellatum* | Glost2 | 0.88 | 0 | 0 | 0 | 0 |
| *Graphis scripta* | Grascr1 | 4.31 | 5.94 | 23.55 | 0.4 | 0.74 |
| *Hortaea acidophila* | Horac1 | 0.29 | 4.5 | 32.87 | 0 | 1.66 |
| *Hortaea thailandica* | Horth1 | 1.84 | 9.8 | 46.55 | 0.2 | 1.85 |
| *Hortaea werneckii* | Horwer1 | 1.32 | 7.85 | 37.07 | 0.4 | 1.47 |
| *Lobaria pulmonaria* | Lobpul1 | 0.06 | 0 | 0 | 0 | 0 |
| *Myriangium duriaei* | Myrdu1 | 0.23 | 5.75 | 20.42 | 0 | 0.81 |
| *Myriangiaceae sp. NC1570* | Myrian1 | 3.9 | 13.41 | 24.85 | 0.8 | 1.08 |
| *Piedraia hortae CBS 480.64 v1.1* | Pieho1_1 | 0.02 | 0 | 0 | 0 | 0 |
| *Polychaeton citri* | Polci1 | 2.32 | 7.31 | 33.77 | 0.1 | 1.46 |
| *Pseudocercospora fijiensis* | Mfijiensis_v2 | 59.25 | 29.42 | 40.44 | 40.7 | 1.52 |
| *Pseudocercospora eumusae* | Myceu1 | 36.49 | 9.57 | 40.9 | 4.4 | 1.64 |
| *Pseudocercospora musae* | Psemus1 | 52.5 | 11.13 | 36.2 | 8.4 | 1.59 |
| *Pseudocercospora ulei* | Pseule1 | 60.76 | 9.65 | 47.32 | 36.4 | 1.28 |
| *Rachicladosporium sp. CCFEE 5018* | Rac5018_1 | 0.4 | 6.33 | 40.2 | 0 | 1.67 |
| *Rachicladosporium antarcticum CCFEE 5527* | Racan1 | 0.6 | 7.13 | 43.46 | 0.1 | 1.72 |
| *Sphaerulina musiva* | Sepmu1 | 11.31 | 15.04 | 41.73 | 2.8 | 1.53 |
| *Sphaerulina populicola* | Seppo1 | 28.45 | 12.71 | 43.84 | 4.7 | 1.51 |
| *Teratosphaeriaceae sp. NC1134* | TerNC1134_1 | 7.84 | 14.24 | 31.67 | 1.1 | 1.61 |
| *Teratosphaeria nubilosa* | Ternu1 | 7.49 | 9.15 | 27.07 | 1.1 | 1.35 |
| *Viridothelium virens* | Tryvi1 | 1.52 | 7.17 | 16.61 | 0 | 0.71 |
| *Lasallia pustulata (Umnilicaria pustulata)* | Umbpus1 | 0.43 | 0 | 0 | 0 | 0 |
| *Xylona heveae* | Xylhe1 | 0.38 | 4.83 | 17.14 | 0 | 0.6 |
| *Zasmidium cellare* | Zasce1 | 1.76 | 6.44 | 37.2 | 0.1 | 1.58 |
| *Zymoseptoria ardabilia* | Zymar1 | 6.32 | 7.45 | 41.2 | 0.3 | 1.66 |
| *Zymoseptoria brevis* | Zymbr1 | 13.97 | 7.48 | 40.56 | 0.3 | 1.68 |
| *Zymoseptoria pseudotritici* | Zymps1 | 8.46 | 8.26 | 43.46 | 0.5 | 1.35 |
| *Zymoseptoria tritici* | Zymtr1 | 17.62 | 11.17 | 43.43 | 6.2 | 1.46 |


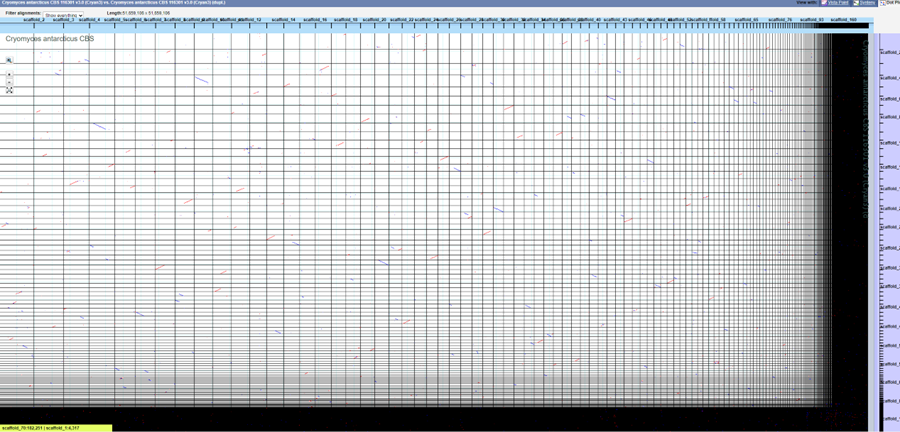


Suppl. Figure 1. VISTA dot plot of *Cryomyces antarcticus* contigs against themselves based on the whole-genome DNA alignment (Dubchak, 2007). The red and blue diagonal lines are nucleotide matches between sequences on different contigs, indicating DNA duplication. The main diagonal of self alignment has been removed for clarity. A higher quality version of the supplementary figure can be accessed at <https://mycocosm.jgi.doe.gov/vista_embed/?viewMode=dotPlot&organism=Cryan3&?&run=8474-crZbaJ90&xdset=3156&ydset=3714&cutoff=1000>


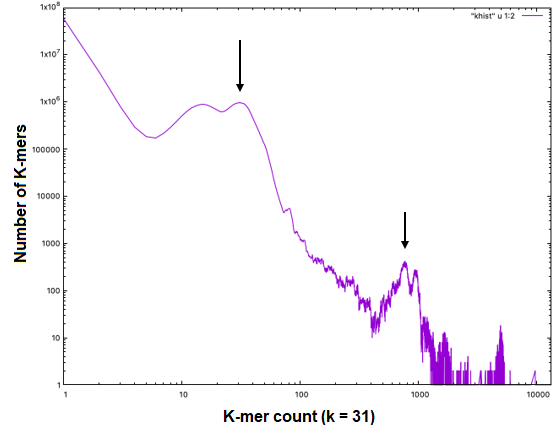


Suppl. Figure 2. K-mer frequency plot of the circular consensus sequencing data from *Cryomyces antarcticus* using a k-mer length of 31 nucleotides. Two peaks indicating diploidy or dikaryosis are shown: a taller peak with high-frequency k-mers and a smaller peak with low-frequency k-mers that are unique to one heterologous chromosome. The other small peaks possibly indicate repeats in the genome.
